# CSCAHHO: Chaotic hybridization algorithm of the Sine Cosine with Harris Hawk optimization algorithms for solving global optimization problems

**DOI:** 10.1371/journal.pone.0263387

**Published:** 2022-05-19

**Authors:** Yu-Jun Zhang, Yu-Xin Yan, Juan Zhao, Zheng-Ming Gao

**Affiliations:** 1 School of Electronics and Information Engineering, Jingchu University of Technology, Jingmen, China; 2 Academy of Arts, Jingchu University of Technology, Jingmen, China; 3 School of Computer Engineering, Jingchu University of Technology, Jingmen, China; 4 Institute of Intelligent Information Technology, Hubei Jingmen Industrial Technology Research Institute, Jingmen, China; Torrens University Australia, AUSTRALIA

## Abstract

Because of the No Free Lunch (NFL) rule, we are still under the way developing new algorithms and improving the capabilities of the existed algorithms. Under consideration of the simple and steady convergence capability of the sine cosine algorithm (SCA) and the fast convergence rate of the Harris Hawk optimization (HHO) algorithms, we hereby propose a new hybridization algorithm of the SCA and HHO algorithm in this paper, called the CSCAHHO algorithm henceforth. The energy parameter is introduced to balance the exploration and exploitation procedure for individuals in the new swarm, and chaos is introduced to improve the randomness. Updating equations is redefined and combined of the equations in the SCA and HHO algorithms. Simulation experiments on 27 benchmark functions and CEC 2014 competitive functions, together with 3 engineering problems are carried out. Comparisons have been made with the original SCA, HHO, Archimedes optimization algorithm (AOA), Seagull optimization algorithm (SOA), Sooty Tern optimization algorithm (STOA), Arithmetic optimizer (AO) and Chimp optimization algorithm (ChOA). Simulation experiments on either unimodal or multimodal, benchmark or CEC2014 functions, or real engineering problems all verified the better performance of the proposed CSAHHO, such as faster convergence rate, low residual errors, and steadier capability. Matlab code of this algorithm is shared in Gitee with the following address: https://gitee.com/yuj-zhang/cscahho.

## 1 Introduction

Thanks to the development of science and technology, we human are now facing more and more complicated and detailed world. More and more functions we formulated to describe the problems in real world are difficult to find the solutions. In order to obtain convinced solutions, we have developed a new kind of algorithms, called nature-inspired algorithms [1], to find the best solutions with random access. But there is a famous theory: no free lunch (NFL) [2], there would be a point balancing the capability and complexity. And we are all busy with finding the best way to solve the problems with easy. Lots of algorithms have been proposed by now, most of them could be classified into four types: evolutionary, human-based, physics-based, and swarm-based algorithms [3], as shown in Table 1.

**Table 1:** Classification of meta-heuristic algorithms.

Classes	Algorithms
**Evolutionary**	Tree Growth Algorithm (TGA) [4] Biogeography-Based Optimizer (BBO) [5] Arithmetic Optimization Algorithm (AOA) [6] Genetic Algorithm (GA) [7] Differential evolution (DE) [8] Genetic Programming (GP) [9] Evolutionary Strategies (ES) [10] Backtracking Search Optimization Algorithm (BSA) [11]
**Human-based**	Teaching based learning algorithm (TBLA) [12] Harmony Search (HS) [13] Imperialist Competitive Algorithm (ICA) [14] Fireworks Algorithm (FWA) [15] Collective Decision Optimization (CSO) [16] Socio Evolution & Learning Optimization Algorithm (SELOA) [17] Child Drawing Development Optimization Algorithm (CDDO) [18] Political Optimizer (PO) [19]
**Physics-based**	Henry Gas Solubility Optimization (HGSO) [20] Big Bang–Big Crunch (BBBC) [21] Multi-verse Optimizer (MVO) [22] Electromagnetic Field Optimization (EFO) [23] Gravitational Search Algorithm (GSA) [24] Thermal Exchange Optimization (TEO) [25] Central Force Optimization (CFO) [26] Farmland Fertility (FF) [27] Vortex Search Algorithm (VSA) [28]
**Swarm-based**	Artificial Bee Colony (ABC) [29] Cuckoo Search (CS) [30] Symbiotic Organisms Search [31] Spotted Hyena Optimizer [32] Lion Optimization Algorithm (LOA) [33] Particle Swarm Optimization (PSO) [34] Firefly Algorithm (FA) [35] Moth Flame Optimization (MFO) [36] Ant Colony Optimization (ACO) [37] Marine Predators Algorithm (MPA) [38] Slime Mould Algorithm (SMA) [39] Grasshopper Optimization Algorithm (GOA) [40] Golden Eagle Optimizer (GEO) [41] Chimp optimization algorithm (ChOA) [42] Sooty Tern Optimization Algorithm (STOA) [43] Seagull optimization algorithm (SOA) [44] Aquila Optimizer (AO) [45] Remora optimization algorithm (ROA) [46] African Vulture Optimization Algorithm (AVOA) [47] Gorilla Troops Optimization Algorithm (GTO) [48]

Although lots of algorithms have been proposed in literature, lots of problems [49] remain being difficult to solve [50]. Therefore, we are still under the way to find more capable algorithms, even improvements to increase the capability slightly.

Considering the simple and better performance of the sine cosine algorithm (SCA) [51], and the fast convergence capability of the Harris Hawk optimization (HHO) [52] algorithm, we propose a hybridized algorithm with chaos, called chaotic hybridization of the SCA and HHO algorithm (CSCAHHO) in this paper. The new algorithm would embrace the advantages of the two algorithms and better performance would be promised.

The rest of this paper would be scheduled as follows: in Section 2, literal review would be made on the algorithms and defects would be discussed. The CSCAHHO algorithm would be proposed in Section 3 and simulation experiments would be carried out in Section 4. Discussions would be made and conclusions would be drawn in Section 5.

## 2 Preliminaries

### 2.1 The SCA and its defects

The SCA is very simple, individuals in the SCA swarms would choose an equation to update their positions randomly. And the equations are related to sine and cosine functions, as formulated as follows:

$$X(t+1)=X(t)+r_{1}\times \sin(r_{2})\times |r_{3}P_{b}(t)-X(t)|$$ (1)

$$X(t+1)=X(t)+r_{1}\times \cos\left(r_{2}\right)\times |r_{3}P_{b}(t)-X(t)|$$ (2)

$$r_{1}=a(1-\frac{t}{maxIter})$$ (3)

Where X(t+1) and *X*(*t*) represent the positions of individuals in the *t* and *t*+1 iteration. *r*_2_ is a random number in an interval of 0 and 2*π*, while *r*_3_ are random numbers in an interval of 0 and 1. *P*_*b*_(*t*) represents the best position at the current iteration *t*. *a* is a constant number and *maxIter* represents the maximum allowed iteration number.

Another random parameter *r*_7_ would be involved to balance the way, if *r*_7_≥0.5, then Eq (1) would be chosen as the formulation to update the positions of individuals, otherwise, Eq (2) would be followed by the individuals to update their positions.

Apparently, the SCA is very easy to understand and coded in applications. The SCA has been very popular just since its birth, and it has been applied in every field in engineering. Li et al. [53] applied the SCA algorithm in solving time series forecasting problems. Reddy et al. [54] solved the unit optimization problem in the electricity market with the SCA algorithm. Nayak et al. [55] made a medical diagnosis system based on the SCA algorithm.

However, the original SCA is so simple that the individuals could only follow their current positions and sine or cosine functionalized distance between them and the global best candidate, which would result in poor global exploration capability and exploitation performance. To increase the capability, Chen et al. introduced the orthogonal learning, multi-swarm, and greedy selection mechanisms to the SCA algorithm, and the improved variant is called OMGSCA [56]. Wang et al. proposed a variant SCA algorithm, various strategies such as Cauchy mutation operator, chaotic local search, and opposition-based learning strategy are introduced [57]. Zhu et al. introduced orthogonal learning to the SCA algorithm [58] and consequently, the basic optimization capabilities of the SCA algorithm are enhanced.

### 2.2 The HHO algorithm and its defects

The HHO algorithm was proposed in 2014 [52], it might be the first nature-inspired algorithms that introduce multiple updating discipline [59] for individuals in swarms to update their positions. Four special conditions were considered and four types of updating ways were involved, individuals in the HHO swarms would choose a way from the four based on the escaping energy *E* and the randomness.

#### a) Exploration procedure

When the escaping energy *E* of the rabbit is larger than 1 or smaller than -1, individuals in the HHO swarm would explore the whole area quickly, two strategies would be adopted to do so, which are formulated as follows:

$$X(t+1)=\left\{ \begin{array}{cc} X_{rand}(t)-r_{1}|X_{rand}(t)-2r_{2}X(t)| & q\geq 0.5\\ P_{b}(t)-X_{m}(t)-r_{3}(LB+r_{4}(UB-LB)) & q<0.5 \end{array}\right.$$ (4)

Where, *X*_*rand*_(*t*) is a random selected candidate at the current iteration, and *X*_*m*_(*t*) represents the averaged position of all of individuals at the current iteration and is calculated with Eq (5). *q* is a random number in an interval of 0 and 1. And [LB, UB] is the definitional domain of the given problem.


$$X_{m}(t)=\frac{1}{N}\sum_{i}^{n}X_{i}(t)$$ (5)

#### b) Exploitation procedure

When a rabbit is found as a prey, individuals in the HHO swarms would perform exploitation procedure according to the status of the rabbits with smart actions. This behavior is controlled by the escaping energy of the rabbits:

$$E=2E_{0}(1-\frac{t}{maxIter})$$ (6)

Where *E*_0_ is the initial energy of the rabbits. When |*E*|≥1, individuals in the HHO swarms would perform exploration while on the contrary, when |*E*|<1, individuals would perform exploitation around the domain and select one way to update their positions based on the real-time escaping energy values and a random number.

*i) Soft besiege*. When *r*≥0.5 and |*E*|≥0.5, individuals in the HHO swarms would be aware that the rabbits keep strong and would run faster to escape, therefore, they would fly around the prey and attack it when possible. This attack method can be expressed by the following formula.

$$X(t+1)=P_{g}(t)-X(t)-E|J∙P_{g}(t)-X(t)|$$ (7)

where *J* represents the ability of the prey to jump randomly and is formulated as:

$$J=2(1-r_{5})$$ (8)

Where *r*_5_ is another random number in (0,1).

*ii) Soft besiege with progressive rapid dives*. When *r*<0.5 and |*E*|≥0.5, the energy of the prey is large enough to escape the capture, so the Harris hawk needs to dive around the prey several times. This behavior can be expressed by the following formula.

$$X(t+1)=\left\{ \begin{array}{cc} Y=P_{g}(t)-E|J∙P_{g}(t)-X(t)| & f(Y)<f(X(t))\\ Z=Y+r_{6}\times LF(D) & f(Z)<f(X(t)) \end{array}\right.$$ (9)

where *r*_6_ is a random vector, and *LF*(*D*) is the Levy flight and calculated as follows:

$$LF(x)=0.01\times \frac{\mu \times \sigma }{{\left|\nu \right|}^{\frac{1}{\beta }}},\sigma ={\left(\frac{\Gamma (1+\beta )\times \sin\left(\frac{\pi \beta }{2}\right)}{\Gamma (\frac{1+\beta }{2})\times \beta \times 2^{\left(\frac{\beta -1}{2}\right)}}\right)}^{\frac{1}{\beta }}$$ (10)

Where *μ* and *ν* are random values fallen in an interval of 0 and 1, and *β* is a default constant number.

*iii) Hard besiege*. When *r*≥0.5 and |*E*|<0.5, individuals in the HHO swarms would perform a hard besiege under consideration of a low escaping energy of the rabbits, they would be eager to catch the prey and the formulation updated their positions would be relevant mainly to the global best position:

$$X(t+1)=P_{g}(t)-E|P_{g}-X(t)|$$ (11)

*iv) Hard besiege with progressive rapid dives*. When *r*<0.5 and |*E*|<0.5, the escape energy of the prey is too low to escape, so the Harris Hawk conducts a hard besiege and finally grabs the prey. This behavior can be expressed by the following formula.


$$X(t+1)=\left\{ \begin{array}{cc} Y=P_{g}(t)-E|J∙P_{g}(t)-X_{m}(t)| & f(Y)<f(X(t))\\ Z=Y+r_{6}\times LF(D) & f(Z)<f(X(t) \end{array}\right.$$ (12)

#### c) Literal review of the HHO algorithm

It was very clear that there were two types of exploration and exploitation procedure for individuals in the HHO swarms to find the global best optima. And under consideration of the escaping energy and a random controlled parameter, individuals in the swarm would carry on four types of exploitation behavior with smart actions. This behavior is not complicated and it could be applied easily with fast convergence rate. Therefore, the HHO algorithm is also applied in solving various kinds of jobs. However, experiments also soon confirmed that individuals in the HHO swarms would be easily been trapped in local optima and the overall results were not promising. So, lots of improvements were made to increase its capability. Zhao et al. introduced piecewise linear mapping to the HHO algorithm, and the phase conversion mechanism of the HHO algorithm is effectively improved by this mapping [60]. Chen et al. [61] proposed a variant of HHO. Chaos, differential evolution strategy and multi-group strategy are introduced by variants. The three improvement strategies have improved different p laces. Experimental results showed that the performance of this variant is quite superior. Al-Betar et al. [62] proposed several improved versions of the HHO algorithm. Improvement strategies include proportional and linear rank-based strategies. Akdag et al. [63] introduced seven kinds of random distribution functions. This variant HHO algorithm is applied to the optimal power flow problem. Fan et al. [64] introduced a quasi-reflective learning mechanism (QRBL). QRBL effectively improves the population diversity and convergence speed of the HHO algorithm. Gupta et al. [65] introduced a variety of strategies. It includes learning based on opposites, nonlinear energy parameters and the setting of strategy of Harris Hawk when catching prey. These strategies improve the exploration efficiency of the HHO algorithm, and can also avoid the occurrence of local optimal. It has also been furthermore applied in solving other global optimization algorithm such as travelling salesman problems [66, 67], multiple objective feature selection problems [68].

## 3 The CSCAHHO algorithm

Individuals in the SCA swarms would update their positions according to their current positions and the random involved sine functionalized distance between them and the global best candidates, and therefore, they approach the global best with lower rate. To increase its speed in convergence, the global best candidate might play more import role, such as the formulations as follows:

$$X(t+1)=P_{g}(t)*LF(D)+r_{1}\times \sin\left(r_{2}\right)\times |r_{3}P_{b}(t)-X(t)|$$ (13)

$$X(t+1)=P_{g}(t)+r_{1}\times \cos\left(r_{2}\right)\times |r_{3}P_{b}(t)-X(t)|$$ (14)

Where LF(D) represents the Levy flights operation with the following equations:

$$LF(x)=0.01\times \frac{\mu \times \sigma }{{\left|\nu \right|}^{\frac{1}{\beta }}},\sigma ={\left(\frac{\Gamma (1+\beta )\times \sin\left(\frac{\pi \beta }{2}\right)}{\Gamma (\frac{1+\beta }{2})\times \beta \times 2^{\left(\frac{\beta -1}{2}\right)}}\right)}^{\frac{1}{\beta }}$$ (15)

On the other hand, if the positions were updated as Eqs (13) and (14), the global best candidates would play too much role in updating that individuals in swarms would be easily trapped in local optima. Therefore, more efforts would be included to make the individuals more diverse and the multiple updating ways of individuals in the HHO algorithms might be suitable. We hereby introduce the multiple updating ways for individuals in the HHO swarms to the updating equations further.

**Fig 1:**
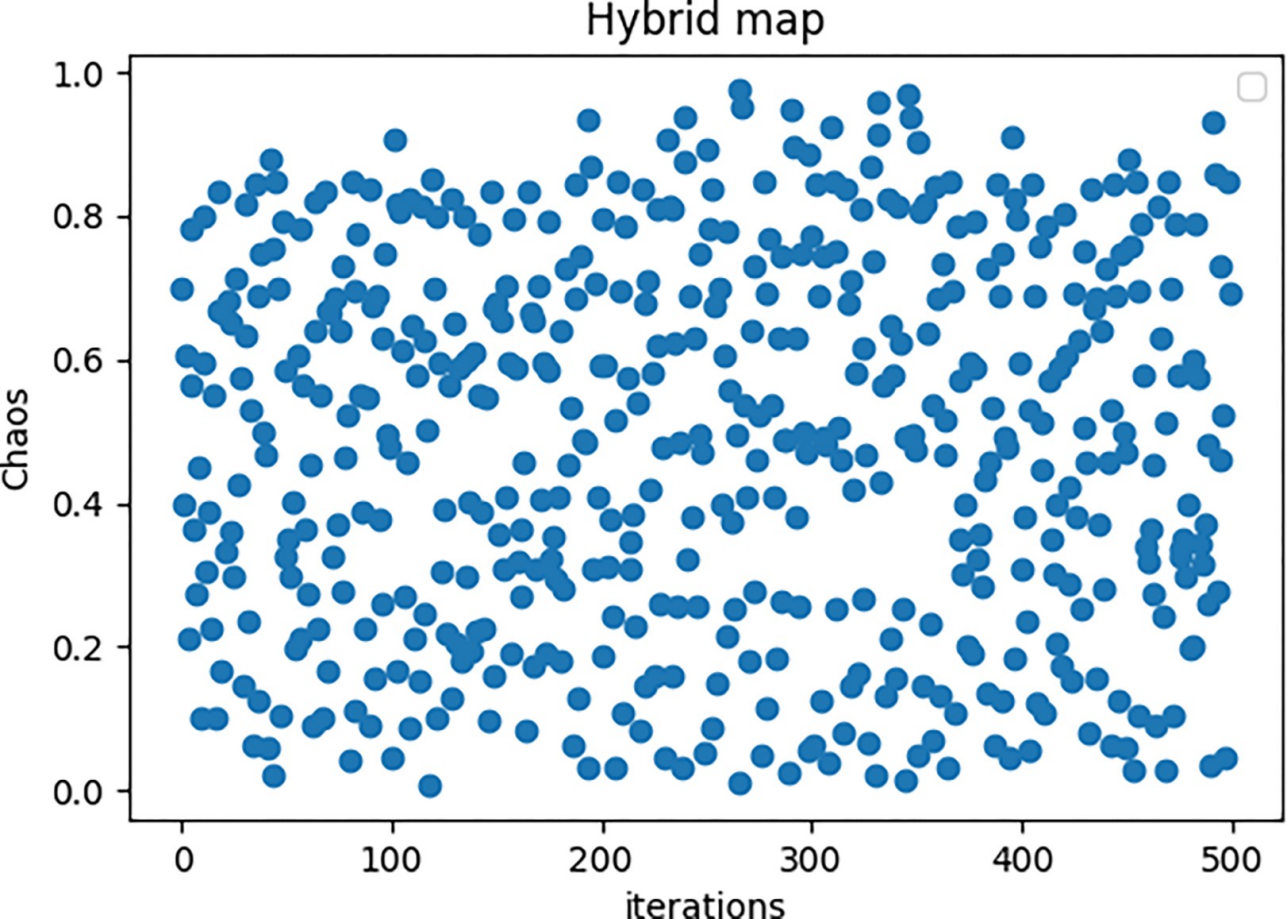
The absolute hybrid chaos.

Furthermore, in order to improve the randomness of the conversion mechanism, the chaotic map which is called Hybrid map is introduced. Due to the characteristics of chaotic mapping, the random performance of the conversion mechanism is well improved. The following formula can be used to express the Hybrid map.

$$C(n+1)=\{\begin{array}{c} b*(1-u_{1}*{C(n)}^{2})\;C(n)<0\\ 1-u_{2}*C(n)\;otherwise \end{array}$$ (16)

where *b* = 0.85, *u*_1_ = 1.8, *u*_2_ = 2.0. The chaos would fluctuate in an interval of -1 and 1, and if the absolute function is introduced, the absolute Hybrid chaos would fluctuate in [0, 1] which is directly the random number generated in computer science with Gauss distribution, as shown in Fig 1.

The formula of the improved conversion mechanism can be expressed as follows.


Ec(n+1)=E+C(n) (17)

**Fig 2:**
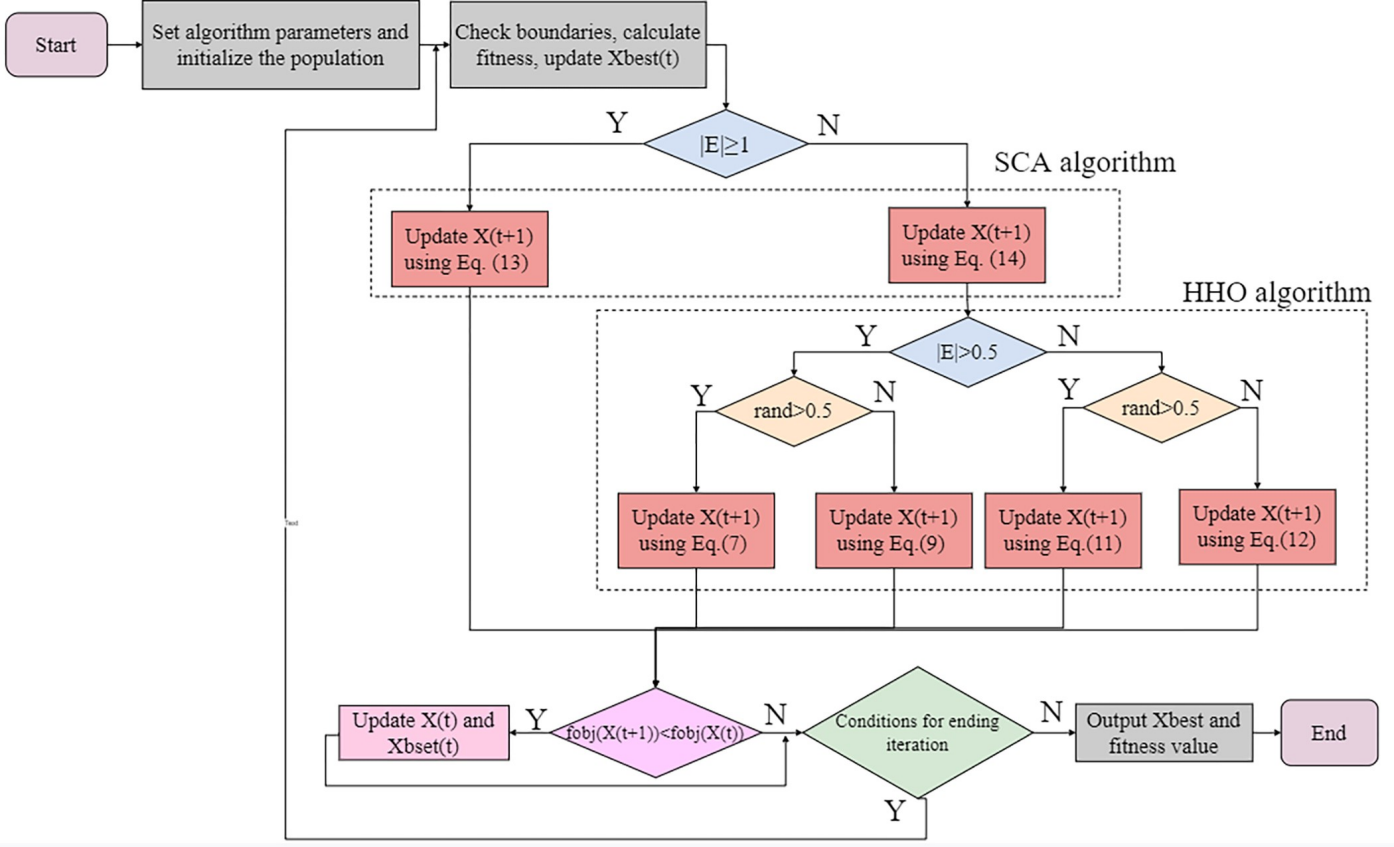
Flowchart of CSCAHHO algorithm.

The flowchart of the CSCAHHO algorithm is shown in Fig 2. The pseudo-code diagram of the CSCAHHO algorithm is given in Table 2.

**Table 2:** Pseudo-code of CSCAHHO algorithm.

Set population size N, the maximum number of iterations T, dimension D Initialize the positions of Individuals *X*_*i*_(*i* = 1,2,…,*N*) **While** (*t*≤*T*) **For** *i* = 1: *N* **If** |*Ec*|≥1 **then** Update the position of X(t+1) using Eq (13) **Else** Update the position of X(t+1) using Eq (14) **If** |*Ec*|≥0.5 *and r*≥0.5 **then** Update the position of X(t+1) using Eq (7) **End if** **If** |*Ec*|≥0.5 *and r*<0.5 **then** Update the position of X(t+1) using Eq (9) **End if** **If** |*Ec*|<0.5 *and r*≥0.5 **then** Update the position of X(t+1) using Eq (11) **End if** **If** |*Ec*|<0.5 *and r*<0.5 **then** Update the position of X(t+1) using Eq (12) **End if** **Memory saving** **End if** **End For** **For** *i* = 1: *N* Check boundaries Calculate the fitness of X(t) Update X_best_(t) **End For** *t* = *t*+1 **End While** **Return** X_best_(t)

The proposed algorithm as shown in **Fig 2** would guarantee individuals in swarms having much larger exploitation ratio at the end stage, and embracing more ways to update their positions. Better performance might be expected with only a few complexities increased, the complexity remains O(T∙N∙D+N) Where T, N, D represent the maximum iterations, number of individuals in swarms, and the dimensionality respectively, as shown in Table 2.

## 4 Simulation experiments

In this section, we would carry on some simulation experiments to verify the capability of the hybridization algorithm. First of all, benchmark function would be introduced to do so. 27 benchmark functions would be involved including 9 unimodal functions (As shown in Table 3), 9 two-dimensional multimodal functions (As shown in Table 4), and 9 multi-dimensional multimodal functions (As shown in Table 5). All of the simulation experiments would be carried out with HP DL380 Gen 10 server with 32GB RAM and Intel Xeon Bronze 3106×2 cores, and Matlab 2017b softwares.

**Table 3:** Unimodal benchmark functions.

Function	Description	Dimensions	Range	f_min_
F1	$f(x)=\sum_{i=1}^{D}x_{i}^{2}$	30,60,100,300,500	[–100,100]	0
F2	$f(x)=\sum_{i=1}^{D}\left|x_{i}\right|$	30,60,100,300,500	[–100,100]	0
F3	$f(x)={\left(10\right)}^{6\frac{i-1}{D-1}}\times \sum_{i=1}^{D}x_{i}^{2}$	30,60,100,300,500	[–100,100]	0
F4	$f(x)=\sum_{i=1}^{D}\left(x_{i+1}^{2}*x_{i}^{2}\right)$	30,60,100,300,500	[–100,100]	0
F5	$f(x)={max}_{i}\{|x_{i}|,1\leq i\leq D\}$	30,60,100,300,500	[–100,100]	0
F6	$f(x)=\sum_{i=1}^{D}\left|x_{i}\right|+\prod_{i=1}^{D}\left|x_{i}\right|$	30,60,100,300,500	[–100,100]	0
F7	$f(x)=\sum_{i=1}^{D}{ix}_{i}^{2}$	30,60,100,300,500	[–100,100]	0
F8	$f(x)=\sum_{i=1}^{D}{\left(\sum_{j=1}^{i}x_{j}\right)}^{2}$	30,60,100,300,500	[–100,100]	0
F9	$f(x)=\sum_{i=1}^{D}{ix}_{i}^{4}$	30,60,100,300,500	[–100,100]	0

**Table 4:** Two-dimensional multimodal functions.

Function	Description	Dimensions	Range	f_min_
F10	$f(x)=7x_{1}^{2}-6\sqrt{3}x_{1}x_{2}+13x_{2}^{2}$	2	[–100,100]	0
F11	$f(x)=195.6316-200e^{\left(-0.02\sqrt{x_{1}^{2}+x_{2}^{2}}\right)}+5e^{\left(\cos3x_{1}+sin3x_{2}\right)}$	2	[–100,100]	0
F12	$f(x)={\left(x_{1}^{2}+x_{2}^{2}\right)}^{0.25}\times (1+sin{\left(50{\left(3x_{1}^{2}+x_{2}^{2}\right)}^{0.1}\right)}^{2})$	2	[–100,100]	0
F13	$f(x)=0.5+\frac{\left({sin(\sqrt{x_{1}^{2}+x_{2}^{2}})}^{2}-0.5\right)}{{\left(1+0.001(x_{1}^{2}+x_{2}^{2})\right)}^{2}}$	2	[–5,5]	0
F14	$f(x)=0.1+{sin(x_{1})}^{2}+{sin(x_{2})}^{2}-0.1e^{\left(-x_{1}^{2}-x_{2}^{2}\right)}$	2	[–10,10]	0
F15	$f(x)=(sin4-\frac{\sin\left(2\sqrt{x_{1}^{2}+x_{2}^{2}}\right)}{2}+\frac{\sin\left(3\sqrt{x_{1}^{2}+x_{2}^{2}}\right)}{3}-\frac{\sin\left(4\sqrt{x_{1}^{2}+x_{2}^{2}}\right)}{4}+4)$ $\times (\sqrt{x_{1}^{2}+x_{2}^{2}}\frac{2}{\sqrt{x_{1}^{2}+x_{2}^{2}}+1})\times (\frac{1}{2}\cos\left(2(argtan\frac{x_{2}}{x_{1}}-\pi )-\frac{1}{2}\right)$ $+\cos\left(argtan\frac{x_{2}}{x_{1}}-\pi \right)+2)$	2	[–100,100]	0
F16	$f(x)=2x_{1}^{2}-1.05x_{1}^{4}+\frac{x_{1}^{6}}{6}+x_{1}x_{2}+x_{2}^{2}$	2	[–100,100]	0
F17	$f(x)=0.26(x_{1}^{2}+x_{2}^{2})-0.48x_{1}x_{2}$	2	[–100,100]	0
F18	$f(x)=0.5+\frac{\left(({sin(\sqrt{x_{1}^{2}+x_{2}^{2}})}^{2})-0.5\right)}{{\left(1+0.001(x_{1}^{2}+x_{2}^{2})\right)}^{2}}$	2	[–20,20]	0

**Table 5:** Multi-dimensional multimodal functions.

Function	Description	Dimensions	Range	f_min_
F19	$f(x)=1+(\sum_{i=1}^{D}{\sin\left(x_{i}\right)}^{2}-e^{\left(-\sum_{i=1}^{D}x_{i}^{2}\right)})\times e^{\left(-\sum_{i=1}^{D}{sin(\sqrt{\left|x_{i}\right|})}^{2}\right)}$	30,60,100,300,500	[–100,100]	0
F20	$f(x)=\sum_{i=1}^{D}x_{i}^{6}(2+sin(\frac{1}{x_{i}}))$	30,60,100,300,500	[–100,100]	0
F21	$f(x)=\sum_{i=1}^{D-1}\left[100{\left(x_{i+1}-x_{i}^{2}\right)}^{2}+{\left(x_{i}-1\right)}^{2}\right]$	30,60,100,300,500	[–100,100]	0
F22	$f(x)=\sum_{i=1}^{D}\left|x_{i}\sin\left(x_{i}\right)+0.1x_{i}\right|$	30,60,100,300,500	[–100,100]	0
F23	$f(x)=\frac{D}{10}+\sum_{i=1}^{D}x_{i}^{2}-\frac{\sum_{i=1}^{D}cos(5\pi x_{i})}{10}$	30,60,100,300,500	[–100,100]	0
F24	$f(x)=1+\frac{\sum_{i=1}^{D}x_{i}^{2}}{4000}-\prod_{i=1}^{D}cos(\frac{x_{i}}{\sqrt{i}})$	30,60,100,300,500	[–100,100]	0
F25	$f(x)=1-cos(2\pi \sqrt{\sum_{i=1}^{D}x_{i}^{2}}+0.1\sqrt{\sum_{i=1}^{D}x_{i}^{2}})$	30,60,100,300,500	[–100,100]	0
F26	$f(x)=10D+\sum_{i=1}^{D}\left[x_{i}^{2}-10cos(2\pi x_{i}\right)]$	30,60,100,300,500	[–100,100]	0
F27	$f(x)=-20e^{\left(-2\sqrt{\frac{\sum_{i=1}^{D}x_{i}^{2}}{D}}\right)}-e^{\left(\frac{\sum_{i=1}^{D}\cos\left(2\pi x_{i}\right)}{D}\right)}+20+e$	30,60,100,300,500	[–100,100]	0

### 4.1 Experiments setup

In order to prove the capability of the improved hybridized algorithm, both the original SCA and HHO algorithms would be involved in simulation experiments. Furthermore, other famous optimization algorithms such as the AO, STOA, SOA, ChOA, and AOA would also be involved. In order to maintain a same condition, the population size, which is the number of individuals in swarms, would be set 30 and the dimension is 30 for all of the swarms. Final results would be the average over 1000 Monte Carlo simulation experiments. All of the other parameters would be set according to the original version of the algorithms respectively, as shown in Table 6.

**Table 6:** Parameter settings for the comparative algorithms.

Algorithm	Parameters
CSCAHHO	a = 2; r∈[0, 1]; r1∈[0, 1]; r2∈[0, 2*π*]; r3∈[0, 2]; E1∈[2, 0]
SCA	r1∈[1, 0]; r2∈[0, 2*π*]; r3∈[0, 2]
AO	U = 0.00565; r1 = 10; ω = 0.005; α = 0.1; δ = 0.1; G1∈[–1, 1]; G2∈[2, 0]
HHO	q∈[0, 1]; r∈[0, 1]; E0∈[–1, 1]; E1∈[2, 0]; E∈[–2, 2];
STOA	Sa∈[2, 0]; r1∈[0.5, 0]; r2∈[0.5, 0]; b = 1
SOA	Fc∈[2, 0]; r1∈[1, 0]; r2∈[1, 0]; b = 1
ChOA	f∈[2, 0]; r1∈[0, 1]; r2∈[0, 1]
AOA	r1∈[1, 0]; r2∈[1, 0]; r3∈[1, 0]

### 4.2 Qualitative experiments

**Fig 3:**
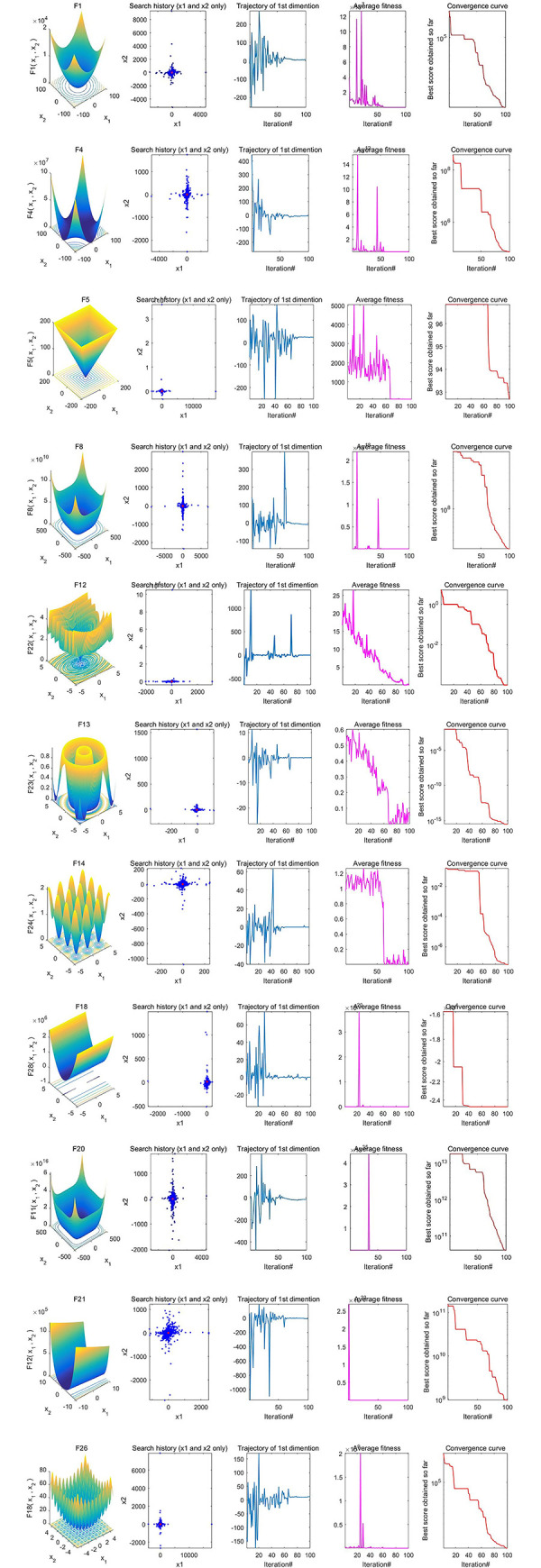
Parameter space, search history, trajectory, average fitness, and convergence curve of CSCAHHO.

First of all, we carried out the qualitative analysis on the CSCAHHO algorithm, search history, trajectory of the first dimension, average fitness values and the convergence curve were shown in **Fig 3**.

Fig 3 shows that the hybridized CSCAHHO algorithm could perform well in optimization on either unimodal or multimodal benchmark functions. Individuals would quickly approach the global best position and find the global optima. The trajectories fluctuate at first but will converge very fast. To be noticed that some of the average fitness values increased during iterations, which meant that individuals in swarms would not always approach the global best and consequently perform well in diversification. The overall fast convergence curve of the best fitness value also verifies such conclusion.

### 4.3 Intensification capability experiments

Unimodal benchmark functions have only one global optima overall their domain. The quicker individuals approach the global best optima; the faster convergence algorithms would achieve. Experiments on unimodal benchmark functions would show the intensification of convergence, and the results were listed in Table 7.

**Table 7:** Unimodal function results, dimension = 30.

Fun	Items	CSCAHHO	SCA	HHO	AOA	SOA	STOA	AO	ChOA
F1	Worst	**0.00000E+00**	1.36079E-01	2.50078E-184	2.72236E+02	1.93156E-26	3.02001E-17	1.14893E-208	2.53106E-13
	Average	**0.00000E+00**	1.53295E-02	1.13904E-185	2.46361E+01	1.65790E-27	4.52581E-18	4.59573E-210	1.30489E-14
	Best	**0.00000E+00**	1.47120E-07	4.19990E-206	3.02682E+00	1.29156E-29	7.93043E-21	1.53558E-300	4.00957E-23
	Middle	**0.00000E+00**	5.30077E-04	6.99086E-196	8.93498E+00	2.50931E-28	9.41940E-19	2.40428E-291	4.62412E-16
	Std	0.00000E+00	3.71163E-02	0.00000E+00	5.36707E+01	4.15174E-27	8.41795E-18	0.00000E+00	5.03917E-14
F2	Worst	**1.49159E-274**	6.39576E-04	9.80034E-93	1.19653E+01	6.32895E-16	1.59786E-10	7.90159E-97	2.39869E-09
	Average	**5.96637E-276**	8.84391E-05	3.92540E-94	6.93707E+00	1.64730E-16	2.20276E-11	3.16080E-98	4.06933E-10
	Best	**2.09097E-299**	2.46496E-07	8.11868E-109	3.25333E+00	9.86383E-18	8.45855E-14	2.04700E-153	2.44373E-18
	Middle	**5.82304E-291**	2.37879E-05	1.02406E-100	7.15369E+00	6.24509E-17	1.12072E-11	7.71196E-147	1.51540E-10
	Std	**0.00000E+00**	1.52378E-04	1.95996E-93	2.23960E+00	1.88568E-16	3.24632E-11	1.58031E-97	6.27569E-10
F3	Worst	**0.00000E+00**	4.57091E+06	2.05981E-172	9.10075E+08	1.71851E-19	2.40362E-09	2.52582E-193	2.73487E-05
	Average	**0.00000E+00**	4.76356E+05	8.27306E-174	2.04339E+08	1.16005E-20	2.60659E-10	1.01033E-194	1.63596E-06
	Best	**0.00000E+00**	4.69554E+01	4.64370E-199	2.63982E+07	2.33274E-23	1.18002E-13	9.88026E-302	9.38527E-14
	Middle	**0.00000E+00**	6.08514E+03	3.26855E-188	1.19755E+08	1.77601E-21	2.07420E-11	7.08115E-276	8.02700E-09
	Std	0.00000E+00	1.05929E+06	0.00000E+00	2.13551E+08	3.47967E-20	6.12679E-10	0.00000E+00	5.64345E-06
F4	Worst	0.00000E+00	5.78482E-14	0.00000E+00	4.72263E+00	5.08834E-59	8.97811E-38	0.00000E+00	4.53912E-34
	Average	0.00000E+00	2.44841E-15	0.00000E+00	2.44776E-01	2.23850E-60	4.38269E-39	0.00000E+00	1.82370E-35
	Best	0.00000E+00	2.35396E-28	0.00000E+00	8.59839E-48	1.45863E-69	1.76605E-47	0.00000E+00	1.24898E-70
	Middle	0.00000E+00	1.63620E-19	0.00000E+00	5.62932E-11	9.89460E-65	1.98453E-42	0.00000E+00	6.19835E-46
	Std	0.00000E+00	1.15473E-14	0.00000E+00	9.68270E-01	1.01817E-59	1.81388E-38	0.00000E+00	9.07663E-35
F5	Worst	**3.26227E-220**	4.02711E+01	1.23369E-92	7.45915E+01	2.58129E-06	5.50621E-05	2.31126E-97	9.28427E-03
	Average	**1.30491E-221**	1.99616E+01	7.91040E-94	2.89781E+01	1.09239E-07	1.40192E-05	9.24521E-99	2.47472E-03
	Best	**3.14766E-250**	3.48556E+00	7.03036E-107	3.72672E+00	6.32774E-11	2.20176E-06	1.66068E-152	2.92318E-05
	Middle	**2.22169E-236**	2.05862E+01	2.88948E-98	2.41152E+01	3.57443E-09	8.92205E-06	2.38083E-145	1.29266E-03
	Std	**0.00000E+00**	8.23404E+00	2.55948E-93	1.92071E+01	5.15078E-07	1.32809E-05	4.62251E-98	3.04025E-03
F6	Worst	**2.70138E-279**	2.52264E-04	1.45297E-91	1.57816E+00	8.28288E-17	3.64855E-11	2.31871E-97	6.73363E-09
	Average	**1.14766E-280**	4.12726E-05	6.08905E-93	2.11876E-01	1.98068E-17	7.43304E-12	9.27482E-99	5.82460E-10
	Best	**2.34864E-302**	2.34974E-07	5.21885E-107	1.24148E-08	9.15944E-2	1.57888E-13	2.22210E-148	6.87403E-16
	Middle	**1.74244E-289**	8.86930E-06	3.38224E-100	2.87969E-02	1.48625E-17	3.78509E-1	5.23764E-141	1.44147E-10
	Std	**0.00000E+00**	7.62651E-05	2.90175E-92	4.10704E-01	2.11280E-17	9.52120E-1	4.63741E-98	1.38115E-09
F7	Worst	**0.00000E+00**	3.89709E+00	1.76486E-184	1.51456E+03	1.24865E-25	5.22147E-15	2.01693E-20	5.25464E-10
	Average	**0.00000E+00**	3.86128E-01	7.06936E-186	2.42755E+02	1.37906E-26	3.58124E-16	8.06773E-203	2.11394E-11
	Best	**0.00000E+00**	9.52261E-06	8.85804E-209	2.63546E+01	9.53522E-31	2.38767E-19	8.84984E-303	7.77555E-21
	Middle	**0.00000E+00**	9.62945E-03	3.81311E-194	1.25272E+02	1.77215E-27	6.60419E-17	2.95720E-288	1.18296E-15
	Std	0.00000E+00	9.18981E-01	0.00000E+00	3.23751E+02	2.76774E-26	1.03808E-1	0.00000E+00	1.05069E-10
F8	Worst	0.00000E+00	2.44957E+03	0.00000E+00	5.47022E+04	7.73145E-45	2.50247E-27	0.00000E+00	5.56860E-18
	Average	0.00000E+00	1.09553E+02	0.00000E+00	7.39909E+03	4.96420E-46	2.68815E-28	0.00000E+00	2.24743E-19
	Best	0.00000E+00	3.93844E-06	0.00000E+00	1.17441E+01	2.78349E-51	6.38289E-3	0.00000E+00	3.32048E-31
	Middle	0.00000E+00	1.02761E-01	0.00000E+00	7.44947E+02	1.06969E-47	7.59757E-30	0.00000E+00	2.50134E-24
	Std	0.00000E+00	4.89374E+02	0.00000E+00	1.51339E+04	1.56655E-45	6.40565E-28	0.00000E+00	1.11333E-18
F9	Worst	0.00000E+00	5.40644E+04	0.00000E+00	6.99575E+06	4.99708E-42	1.52857E-26	0.00000E+00	2.53986E-18
	Average	0.00000E+00	3.87692E+03	0.00000E+00	3.19111E+05	2.00496E-43	1.91103E-27	0.00000E+00	1.04636E-19
	Best	0.00000E+00	4.04592E-04	0.00000E+00	2.96558E+01	1.43534E-52	2.30719E-33	0.00000E+00	5.37781E-30
	Middle	0.00000E+00	5.97360E+00	0.00000E+00	3.85847E+03	4.24784E-47	7.66605E-29	0.00000E+00	2.52044E-23
	Std	0.00000E+00	1.29470E+04	0.00000E+00	1.39704E+06	9.99291E-43	4.16199E-27	0.00000E+00	5.07531E-19

Results in Table 7 showed that in most cases, the hybridized CSCAHHO algorithm would perform best, although sometimes other algorithms such as the SCA, HHO, AO would also find the global optima with the same iteration number.

### 4.4 Diversification capability experiments

Multimodal benchmark functions have many local optima with one global optima. Individuals would be easily trapped in local optima when they are approaching. To avoid being trapped, individuals should have diversification capability. Simulation experiments results on multimodal benchmark functions were shown in Tables 8 and 9.

**Table 8:** Results of multimodal two-dimensional function.

Fun	Items	CSCAHHO	SCA	HHO	AOA	SOA	STOA	AO	ChOA
F10	Worst	0.00000E+00	4.42401E-126	3.28471E-221	0.00000E+00	8.67750E-190	4.64295E-114	4.01939E-204	1.33818E-208
	Average	0.00000E+00	1.77300E-127	1.40133E-222	0.00000E+00	3.47104E-191	1.86971E-115	1.61185E-205	5.79136E-210
	Best	0.00000E+00	1.33924E-147	3.83704E-289	0.00000E+00	4.03715E-211	3.54210E-137	8.26063E-304	3.58531E-253
	Middle	0.00000E+00	6.20841E-136	6.09309E-234	0.00000E+00	2.09723E-200	4.55938E-121	1.67105E-287	5.47705E-222
	Std	0.00000E+00	8.84731E-127	0.00000E+00	0.00000E+00	0.00000E+00	9.28343E-115	0.00000E+00	0.00000E+00
F11	Worst	**2.57174E-03**	2.91275E-03	2.57194E-03	1.54999E-02	2.58487E-03	2.58106E-03	3.50430E-03	2.67973E-03
	Average	**2.57174E-03**	2.69699E-03	2.57178E-03	7.98041E-03	2.57509E-03	2.57447E-03	2.85503E-03	2.60463E-03
	Best	**2.57174E-03**	2.57731E-03	2.57174E-03	3.17405E-03	2.57174E-03	2.57189E-03	2.59172E-03	2.57870E-03
	Middle	**2.57174E-03**	2.68124E-03	2.57174E-03	6.83822E-03	2.57315E-03	2.57440E-03	2.76065E-03	2.59011E-03
	Std	**3.15669E-12**	9.19234E-05	6.31701E-08	3.66308E-03	3.97876E-06	2.01079E-06	2.64923E-04	3.10758E-05
F12	Worst	0.00000E+00	4.65101E-35	1.12620E-51	0.00000E+00	6.55068E-50	4.04551E-31	9.91103E-34	8.82996E-68
	Average	0.00000E+00	4.78371E-36	8.95439E-53	0.00000E+00	3.84155E-51	5.10146E-32	3.97923E-35	3.57364E-69
	Best	0.00000E+00	1.05900E-39	6.76820E-59	0.00000E+00	8.06434E-56	2.49250E-36	8.08432E-76	1.49089E-81
	Middle	0.00000E+00	8.97154E-37	3.53689E-56	0.00000E+00	1.06333E-52	3.48897E-33	3.64052E-72	6.45958E-74
	Std	0.00000E+00	1.22094E-35	2.90871E-52	0.00000E+00	1.35100E-50	1.10262E-31	1.98191E-34	1.76520E-68
F13	Worst	0.00000E+00	0.00000E+00	0.00000E+00	0.00000E+00	9.71592E-03	9.71593E-03	1.97508E-06	9.71599E-03
	Average	0.00000E+00	0.00000E+00	0.00000E+00	0.00000E+00	1.55455E-03	2.33182E-03	7.90030E-08	7.25777E-03
	Best	0.00000E+00	0.00000E+00	0.00000E+00	0.00000E+00	0.00000E+00	0.00000E+00	0.00000E+00	0.00000E+00
	Middle	0.00000E+00	0.00000E+00	0.00000E+00	0.00000E+00	0.00000E+00	0.00000E+00	0.00000E+00	9.71591E-03
	Std	0.00000E+00	0.00000E+00	0.00000E+00	0.00000E+00	3.63536E-03	4.23507E-03	3.95015E-07	3.88804E-03
F14	Worst	0.00000E+00	0.00000E+00	0.00000E+00	0.00000E+00	1.00354E-01	1.00087E-01	0.00000E+00	1.00687E-01
	Average	0.00000E+00	0.00000E+00	0.00000E+00	0.00000E+00	2.00183E-02	1.60075E-02	0.00000E+00	5.72139E-02
	Best	0.00000E+00	0.00000E+00	0.00000E+00	0.00000E+00	0.00000E+00	0.00000E+00	0.00000E+00	0.00000E+00
	Middle	0.00000E+00	0.00000E+00	0.00000E+00	0.00000E+00	0.00000E+00	0.00000E+00	0.00000E+00	7.88698E-02
	Std	0.00000E+00	0.00000E+00	0.00000E+00	0.00000E+00	4.08622E-02	3.74342E-02	0.00000E+00	4.71237E-02
F15	Worst	0.00000E+00	1.89130E-12	9.82787E-96	0.00000E+00	4.61227E+00	4.62657E+00	5.20832E-96	4.64876E+00
	Average	0.00000E+00	7.56521E-14	3.94446E-97	0.00000E+00	3.56176E-01	8.84396E-01	2.08333E-97	2.47478E+00
	Best	0.00000E+00	1.41071E-69	1.63017E-109	0.00000E+00	2.73100E-99	1.28403E-60	2.79634E-138	7.44590E-121
	Middle	0.00000E+00	5.48595E-63	6.87231E-104	0.00000E+00	7.57558E-74	4.85483E-51	4.92612E-123	3.91945E+00
	Std	0.00000E+00	3.78261E-13	1.96530E-96	0.00000E+00	1.23362E+00	1.80616E+00	1.04166E-96	2.24923E+00
F16	Worst	0.00000E+00	1.79342E-129	1.12202E-201	0.00000E+00	1.86824E-190	3.72894E-118	5.61879E-195	1.02399E-279
	Average	0.00000E+00	7.17400E-131	5.39142E-203	0.00000E+00	7.47297E-192	1.54431E-119	2.24768E-196	4.09594E-281
	Best	0.00000E+00	1.57802E-151	6.41333E-232	0.00000E+00	8.07670E-217	5.63515E-149	2.02571E-301	0.00000E+00
	Middle	0.00000E+00	1.83902E-142	4.15232E-213	0.00000E+00	5.26547E-207	9.20908E-128	9.05209E-281	5.26346E-306
	Std	0.00000E+00	3.58683E-130	0.00000E+00	0.00000E+00	0.00000E+00	7.45151E-119	0.00000E+00	0.00000E+00
F17	Worst	0.00000E+00	7.59674E-105	1.47002E-236	0.00000E+00	3.78063E-149	8.26449E-94	7.92763E-202	1.17828E-125
	Average	0.00000E+00	3.04092E-106	6.43084E-238	0.00000E+00	2.48657E-150	3.61014E-95	3.17105E-203	9.16430E-127
	Best	0.00000E+00	1.15889E-133	0.00000E+00	0.00000E+00	4.05942E-172	1.37568E-116	0.00000E+00	1.21187E-153
	Middle	0.00000E+00	5.70118E-118	5.38771E-272	0.00000E+00	9.16022E-162	8.69849E-101	3.09806E-292	1.21056E-132
	Std	0.00000E+00	1.51930E-105	0.00000E+00	0.00000E+00	8.79507E-150	1.65099E-94	0.00000E+00	3.10132E-126
F18	Worst	0.00000E+00	0.00000E+00	0.00000E+00	0.00000E+00	9.71594E-03	9.71591E-03	0.00000E+00	9.71597E-03
	Average	0.00000E+00	0.00000E+00	0.00000E+00	0.00000E+00	3.10909E-03	1.55455E-03	0.00000E+00	4.73752E-03
	Best	0.00000E+00	0.00000E+00	0.00000E+00	0.00000E+00	0.00000E+00	0.00000E+00	0.00000E+00	0.00000E+00
	Middle	0.00000E+00	0.00000E+00	0.00000E+00	0.00000E+00	0.00000E+00	0.00000E+00	0.00000E+00	2.99847E-03
	Std	0.00000E+00	0.00000E+00	0.00000E+00	0.00000E+00	4.62570E-03	3.63536E-03	0.00000E+00	4.62254E-03

**Table 9:** Multi-peak multidimensional function results.

Fun	Items	CSCAHHO	SCA	HHO	AOA	SOA	STOA	AO	ChOA
F19	Worst	0.00000E+00	1.00000E+00	0.00000E+00	1.00000E+00	1.00000E+00	1.00000E+00	3.26035E-02	1.00000E+00
	Average	0.00000E+00	1.00000E+00	0.00000E+00	1.00000E+00	1.00000E+00	1.00000E+00	4.45204E-03	1.00000E+00
	Best	0.00000E+00	1.00000E+00	0.00000E+00	1.00000E+00	1.00000E+00	1.00000E+00	0.00000E+00	1.00000E+00
	Middle	0.00000E+00	1.00000E+00	0.00000E+00	1.00000E+00	1.00000E+00	1.00000E+00	0.00000E+00	1.00000E+00
	Std	0.00000E+00	8.27657E-10	0.00000E+00	7.24111E-10	5.91662E-11	4.04979E-11	9.80106E-03	1.06767E-09
F20	Worst	0.00000E+00	1.22672E+08	0.00000E+00	1.19962E+10	1.20510E-54	3.81316E-32	0.00000E+00	4.22292E-20
	Average	0.00000E+00	6.64317E+06	0.00000E+00	5.44664E+08	4.84044E-56	1.56050E-33	0.00000E+00	1.89061E-21
	Best	0.00000E+00	5.67065E-04	0.00000E+00	1.09673E+02	2.66116E-74	4.19241E-42	0.00000E+00	3.89018E-37
	Middle	0.00000E+00	3.81335E+04	0.00000E+00	1.42227E+05	5.03557E-64	1.03209E-37	0.00000E+00	1.87441E-27
	Std	0.00000E+00	2.50270E+07	0.00000E+00	2.39342E+09	2.40980E-55	7.62067E-33	0.00000E+00	8.45098E-21
F21	Worst	2.87055E+01	8.09575E+05	**2.17495E-02**	8.95214E+06	2.88799E+01	2.88086E+01	2.43537E-02	2.89802E+01
	Average	1.03224E+01	3.80103E+04	7.32192E-03	4.32697E+05	2.80818E+01	2.79376E+01	**3.45044E-03**	2.88572E+01
	Best	**8.40020E-09**	2.95155E+01	1.63190E-04	7.05925E+02	2.69061E+01	2.70870E+01	7.21604E-06	2.80821E+01
	Middle	**6.70213E-05**	3.00778E+02	5.80115E-03	2.98951E+04	2.80676E+01	2.80346E+01	9.73596E-04	2.89302E+01
	Std	1.40470E+01	1.61220E+05	6.18817E-03	1.77975E+06	6.92619E-01	6.13582E-01	**5.96755E-03**	1.89763E-01
F22	Worst	3.97394E-05	5.99938E+01	**6.69835E-89**	1.06620E+02	1.02093E-03	2.46237E-03	3.97219E-03	1.17335E-02
	Average	1.58958E-06	1.08752E+01	**2.67934E-90**	3.90342E+01	4.75419E-05	4.72262E-04	1.70960E-04	9.62290E-04
	Best	**2.16990E-301**	1.72484E-03	3.19700E-108	6.17887E+00	9.61730E-18	2.61495E-11	4.93940E-151	3.19173E-09
	Middle	**6.32464E-291**	2.53192E+00	1.55047E-101	2.67171E+01	1.97827E-15	1.50111E-04	1.15963E-126	2.18377E-04
	Std	7.94789E-06	1.66305E+01	**1.33967E-89**	3.01961E+01	2.04534E-04	8.35705E-04	7.94217E-04	2.35037E-03
F23	Worst	0.00000E+00	3.86668E+00	0.00000E+00	2.15089E+02	4.44089E-16	4.88498E-15	0.00000E+00	1.26180E-10
	Average	0.00000E+00	2.83856E-01	0.00000E+00	2.06664E+01	1.77636E-17	1.35003E-15	0.00000E+00	6.04333E-12
	Best	0.00000E+00	1.36929E-05	0.00000E+00	2.88845E+00	0.00000E+00	0.00000E+00	0.00000E+00	4.44089E-16
	Middle	0.00000E+00	8.42230E-03	0.00000E+00	1.11112E+01	0.00000E+00	8.88178E-16	0.00000E+00	4.88498E-15
	Std	0.00000E+00	8.17640E-01	0.00000E+00	4.10063E+01	8.88178E-17	1.07625E-15	0.00000E+00	2.54187E-11
F24	Worst	0.00000E+00	8.62407E-01	0.00000E+00	1.06469E+00	1.43616E-02	1.05156E-01	0.00000E+00	1.03856E-01
	Average	0.00000E+00	1.64327E-01	0.00000E+00	6.56160E-01	1.39059E-03	1.13075E-02	0.00000E+00	2.10747E-02
	Best	0.00000E+00	4.78706E-07	0.00000E+00	3.96963E-01	0.00000E+00	0.00000E+0	0.00000E+00	0.00000E+00
	Middle	0.00000E+00	2.99048E-02	0.00000E+00	6.55694E-01	0.00000E+00	1.11022E-16	0.00000E+00	7.54628E-03
	Std	0.00000E+00	2.31564E-01	0.00000E+00	1.67602E-01	3.91503E-03	2.49053E-02	0.00000E+00	2.92255E-02
F25	Worst	**0.00000E+00**	1.09990E+00	3.14307E-94	1.37162E+01	9.98735E-02	1.99873E-01	5.10741E-02	1.99873E-01
	Average	**0.00000E+00**	3.56189E-01	2.09694E-95	3.93993E+00	9.98734E-02	1.43873E-01	2.70004E-03	1.05691E-01
	Best	**0.00000E+00**	9.99974E-02	1.29586E-105	1.51681E+00	9.98733E-02	9.98733E-02	4.89362E-146	9.98733E-02
	Middle	**0.00000E+00**	2.99903E-01	7.29207E-99	3.29174E+00	9.98733E-02	9.98734E-02	4.27522E-118	9.98734E-02
	Std	**0.00000E+00**	1.95884E-01	7.22646E-95	2.63843E+00	4.34223E-08	5.06623E-02	1.02711E-02	2.09342E-02
F26	Worst	0.00000E+00	1.76861E+02	0.00000E+00	4.94533E+02	1.09093E+01	2.06797E+01	2.98461E-03	6.79419E+01
	Average	0.00000E+00	5.42215E+01	0.00000E+00	2.66044E+02	7.20539E-01	2.76368E+00	1.19384E-04	8.08753E+00
	Best	0.00000E+00	2.13852E-02	0.00000E+00	4.33381E+01	0.00000E+00	1.13687E-13	0.00000E+00	3.60387E-11
	Middle	0.00000E+00	3.94367E+01	0.00000E+00	3.00722E+02	0.00000E+00	1.00054E+00	0.00000E+00	4.06385E+00
	Std	0.00000E+00	5.00737E+01	0.00000E+00	1.25558E+02	2.55362E+00	4.84200E+00	5.96922E-04	1.39045E+01
F27	Worst	8.88178E-16	2.04075E+01	8.88178E-16	1.99852E+01	2.00000E+01	2.00000E+01	8.88178E-16	2.00000E+01
	Average	8.88178E-16	2.02974E+01	8.88178E-16	7.99410E-01	2.00000E+01	2.00000E+01	8.88178E-16	2.00000E+01
	Best	8.88178E-16	2.01354E+01	8.88178E-16	8.88178E-16	2.00000E+01	2.00000E+01	8.88178E-16	2.00000E+01
	Middle	8.88178E-16	2.03085E+01	8.88178E-16	8.88178E-16	2.00000E+01	2.00000E+01	8.88178E-16	2.00000E+01
	Std	0.00000E+00	7.15221E-02	0.00000E+00	3.99705E+00	4.39478E-09	4.96225E-09	0.00000E+00	3.69711E-09

Diversification experiments results shown in Tables 8 and 9 also verified that the proposed improved CSCAHHO algorithm would perform better in most cases. However, some of the other algorithms would also achieve the top prize sometimes.

### 4.5 Acceleration convergence experiments

**Fig 4:**
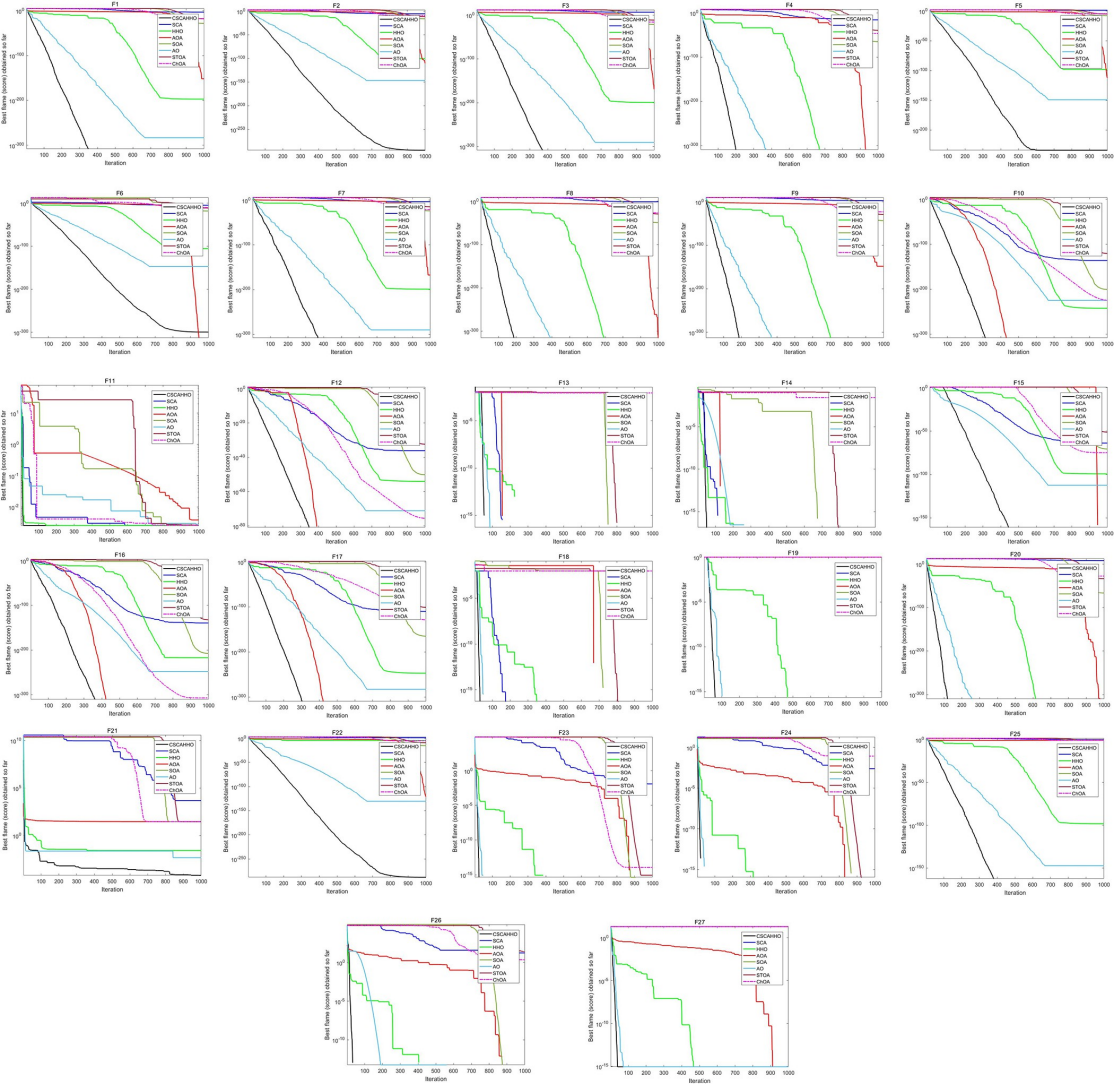
Convergence curves of 27 benchmark functions.

For a better and clear understanding of the capabilities, acceleration convergence analysis was carried out on all of the involved benchmark functions. Results were shown in **Fig 4**.

All of the results demonstrate the better performance of the improved CSCAHHO algorithm. The residual errors are the smallest and the convergence curve are more steadier and the convergence rate are more faster.

### 4.6 Scalability experiments

We are facing more numbers in dimensionality when describing the problems in our real world. Therefore, the capability in solving high dimensional problems is of most important. Scalability experiments would be carried out in this section and the final results were shown in Tables 10–12.

**Table 10:** Test results of the benchmark function (F1-F9, F19-F27), the dimension is fixed to 60.

Fun	Items	CSCAHHO	SCA	HHO	AOA	SOA	STOA	AO	ChOA
F1	Worst	**0.00000E+00**	4.14562E+03	6.04916E-179	9.25677E+04	1.28021E-18	1.52748E-11	6.17785E-204	2.95803E-06
	Average	**0.00000E+00**	4.75731E+02	2.42107E-180	6.78495E+04	2.42921E-19	3.41642E-12	2.47114E-205	1.99431E-07
	Best	**0.00000E+00**	3.20544E-02	5.87121E-216	4.11403E+0	1.88628E-21	1.41074E-14	1.88660E-301	7.86250E-10
	Middle	**0.00000E+00**	1.87536E+02	3.46000E-196	6.97106E+04	8.55300E-20	1.16827E-12	5.22941E-290	2.18137E-08
	Std	0.00000E+00	8.77096E+02	0.00000E+00	1.55053E+04	3.42424E-19	4.42544E-12	0.00000E+00	5.93544E-07
F2	Worst	**4.56303E-273**	1.59740E+01	8.37089E-93	9.35104E+02	1.87473E-11	5.82935E-07	4.88619E-98	1.63649E-04
	Average	**1.82524E-274**	1.32216E+00	5.14073E-94	6.48172E+02	4.66421E-12	4.42052E-08	1.95448E-99	2.71448E-05
	Best	**1.27873E-301**	1.29915E-02	4.73931E-106	3.65583E+02	2.21353E-13	2.26043E-09	3.96259E-154	2.28493E-06
	Middle	**1.58837E-288**	2.51616E-01	2.69381E-100	6.51504E+02	4.02031E-12	1.12393E-08	7.70780E-145	1.49414E-05
	Std	**0.00000E+00**	3.25317E+00	1.86252E-93	1.43731E+02	4.23317E-12	1.14695E-07	9.77239E-99	3.26039E-05
F3	Worst	**0.00000E+00**	1.03257E+11	1.01507E-176	2.88202E+12	1.42628E-10	1.28498E-03	2.54691E-214	9.65589E+00
	Average	**0.00000E+00**	1.30932E+10	7.92479E-178	1.90144E+12	2.06690E-11	1.13063E-04	1.01877E-215	1.77161E+00
	Best	**0.00000E+00**	1.01470E+08	4.16499E-209	8.06745E+11	9.11102E-14	1.12025E-06	4.55596E-296	6.53220E-05
	Middle	**0.00000E+00**	5.51044E+09	1.14057E-186	1.95554E+12	3.61432E-12	1.22323E-05	7.41461E-277	4.36675E-01
	Std	0.00000E+00	2.21125E+10	0.00000E+00	5.14808E+11	3.89960E-11	2.78569E-04	0.00000E+00	2.59210E+00
F4	Worst	0.00000E+00	2.45541E+02	0.00000E+00	6.29025E+06	4.34763E-42	5.26271E-24	0.00000E+00	3.28659E-18
	Average	0.00000E+00	1.05513E+01	0.00000E+00	1.20063E+06	3.93166E-43	2.53035E-25	0.00000E+00	1.31919E-19
	Best	0.00000E+00	4.40256E-08	0.00000E+00	1.27604E+04	1.91680E-48	9.61975E-36	0.00000E+00	1.06753E-32
	Middle	0.00000E+00	4.73842E-03	0.00000E+00	3.69540E+05	1.41675E-44	4.91052E-29	0.00000E+00	6.77329E-26
	Std	0.00000E+00	4.89940E+01	0.00000E+00	1.68748E+06	9.83356E-43	1.05684E-24	0.00000E+00	6.57224E-19
F5	Worst	**1.38324E-218**	8.22181E+01	4.27306E-93	9.63492E+01	3.35702E+01	5.90591E+01	6.00579E-100	3.89764E+00
	Average	**5.53297E-220**	7.03028E+01	3.04034E-94	9.23155E+01	4.40877E+00	5.51746E+00	2.40232E-101	1.04288E+00
	Best	**9.49039E-253**	5.56846E+01	1.32708E-102	8.57735E+01	1.65836E-05	3.33334E-03	2.69407E-152	9.63643E-02
	Middle	**1.36176E-237**	7.03815E+01	1.71631E-97	9.21125E+01	7.39793E-02	2.89830E-01	1.20687E-145	8.52251E-01
	Std	**0.00000E+00**	6.80484E+00	9.14612E-94	2.16417E+00	8.77484E+00	1.35443E+01	1.20116E-100	9.14084E-01
F6	Worst	**1.89762E-276**	1.99637E+00	9.59155E-94	1.43743E+01	3.15778E-12	6.71593E-08	5.18133E-99	1.68239E-05
	Average	**7.64449E-278**	3.84330E-01	3.84849E-95	1.10078E+01	1.09143E-12	1.07196E-08	2.07253E-100	5.12981E-06
	Best	**1.35954E-302**	1.01757E-03	2.85013E-108	7.39455E+00	7.81592E-14	3.13767E-10	1.31130E-152	6.87004E-07
	Middle	**7.31978E-289**	1.07938E-01	5.10903E-102	1.10487E+01	7.73673E-13	9.30326E-09	2.14998E-144	3.64108E-06
	Std	**0.00000E+00**	5.64650E-01	1.91807E-94	1.86313E+00	9.05380E-13	1.33790E-08	1.03627E-99	4.73006E-06
F7	Worst	**0.00000E+00**	3.61619E+04	8.12665E-186	2.02017E+06	4.07528E-17	6.27978E-10	7.32460E-200	1.08991E-04
	Average	**0.00000E+00**	7.92601E+03	4.04783E-187	1.38761E+06	4.91218E-18	1.35262E-10	2.92984E-201	7.02542E-06
	Best	**0.00000E+00**	2.49664E+01	4.59710E-208	9.30621E+05	2.32884E-20	1.03671E-12	3.16194E-303	1.95311E-08
	Middle	**0.00000E+00**	5.54429E+03	2.23002E-193	1.40298E+06	2.57303E-18	5.47113E-11	1.19099E-289	6.58669E-07
	Std	0.00000E+00	9.58585E+03	0.00000E+00	3.06939E+05	8.09504E-18	1.87480E-10	0.00000E+00	2.20866E-05
F8	Worst	0.00000E+00	4.42278E+07	0.00000E+00	6.58924E+08	3.70754E-30	4.75916E-17	0.00000E+00	7.19908E-08
	Average	0.00000E+00	9.31617E+06	0.00000E+00	5.05776E+08	5.83903E-31	5.45964E-18	0.00000E+00	3.69608E-09
	Best	0.00000E+00	1.50712E+04	0.00000E+00	3.02472E+08	6.50436E-35	3.65931E-21	0.00000E+00	1.07601E-16
	Middle	0.00000E+00	6.27875E+06	0.00000E+00	5.00126E+08	2.76046E-31	1.08362E-18	0.00000E+00	4.22995E-11
	Std	0.00000E+00	1.08060E+07	0.00000E+00	9.32541E+07	9.87273E-31	1.19294E-17	0.00000E+00	1.43301E-08
F9	Worst	0.00000E+00	8.64358E+08	0.00000E+00	1.69863E+10	5.18205E-27	1.63320E-15	0.00000E+00	1.40167E-06
	Average	0.00000E+00	1.45427E+08	0.00000E+00	9.94919E+09	2.31732E-28	1.11591E-16	0.00000E+00	6.85076E-08
	Best	0.00000E+00	5.35947E+05	0.00000E+00	2.77752E+09	1.51755E-33	8.02757E-21	0.00000E+00	3.78908E-12
	Middle	0.00000E+00	7.23702E+07	0.00000E+00	9.84470E+09	1.55042E-31	7.50565E-18	0.00000E+00	1.88711E-09
	Std	0.00000E+00	1.92208E+08	0.00000E+00	2.78315E+09	1.03330E-27	3.31367E-16	0.00000E+00	2.79923E-07
F19	Worst	1.00000E+00	1.00000E+00	1.00000E+00	1.00000E+00	1.00000E+00	1.00000E+00	1.00000E+00	1.00000E+00
	Average	8.00000E-01	1.00000E+00	4.00000E-02	1.00000E+00	1.00000E+00	1.00000E+00	2.77344E-01	1.00000E+00
	Best	0.00000E+00	1.00000E+00	0.00000E+00	1.00000E+00	1.00000E+00	1.00000E+00	0.00000E+00	1.00000E+00
	Middle	1.00000E+00	1.00000E+00	0.00000E+00	1.00000E+00	1.00000E+00	1.00000E+00	0.00000E+00	1.00000E+00
	Std	4.08248E-01	9.06493E-17	2.00000E-01	4.53247E-17	0.00000E+00	0.00000E+00	4.21591E-01	4.53247E-17
F20	Worst	0.00000E+00	1.13719E+12	0.00000E+00	1.08422E+13	1.07036E-32	4.24953E-21	0.00000E+00	7.89908E-09
	Average	0.00000E+00	1.11688E+11	0.00000E+00	6.70441E+12	4.34292E-34	5.02614E-22	0.00000E+00	1.18809E-09
	Best	0.00000E+00	2.16946E+09	0.00000E+00	3.51412E+12	9.50607E-48	2.19720E-26	0.00000E+00	8.67346E-19
	Middle	0.00000E+00	4.47255E+10	0.00000E+00	6.69506E+12	1.42848E-40	5.63689E-23	0.00000E+00	1.97496E-11
	Std	0.00000E+00	2.23000E+11	0.00000E+00	1.59989E+12	2.13959E-33	9.18188E-22	0.00000E+00	2.26387E-09
F21	Worst	5.84039E+01	3.84936E+09	1.09336E-01	6.64402E+10	5.88002E+01	5.87953E+01	**1.00186E-01**	5.88917E+01
	Average	2.56965E+01	5.85387E+08	2.06722E-02	4.43437E+10	5.84444E+01	5.83350E+01	**1.45514E-02**	5.86643E+01
	Best	**4.18378E-07**	4.00088E+06	5.17871E-06	2.96395E+10	5.71008E+01	5.71264E+01	3.46866E-06	5.74944E+01
	Middle	**2.93600E-04**	3.65192E+08	8.09638E-03	4.25576E+10	5.86058E+01	5.85559E+01	5.97138E-03	5.88482E+01
	Std	2.95872E+01	7.82138E+08	2.85883E-02	9.07911E+09	4.17474E-01	5.27514E-01	2.33824E-02	**3.88121E-01**
F22	Worst	1.36270E-04	2.14330E+02	**4.75961E-95**	6.12765E+02	4.18756E-03	4.19878E+00	4.69922E-04	2.70062E-02
	Average	5.45081E-06	7.73213E+01	**1.99829E-96**	4.03676E+02	2.77321E-04	1.80542E-01	2.60986E-05	4.30182E-03
	Best	**8.78205E-302**	1.29927E+01	2.11728E-110	2.37139E+02	3.82004E-12	2.29972E-07	3.15456E-152	8.78454E-06
	Middle	**1.17707E-285**	6.96336E+01	5.43005E-100	3.95535E+02	9.45113E-11	1.85327E-03	4.38475E-137	1.78587E-03
	Std	2.72541E-05	4.85879E+01	**9.50455E-96**	1.01169E+02	8.67117E-04	8.37918E-01	9.60975E-05	6.34713E-03
F23	Worst	0.00000E+00	2.60970E+03	0.00000E+00	8.87846E+04	1.77636E-15	5.49554E-10	1.25233E-13	8.05780E-06
	Average	0.00000E+00	4.24835E+02	0.00000E+00	6.56710E+04	1.13687E-15	5.15267E-11	5.00933E-15	1.14623E-06
	Best	0.00000E+00	9.82029E-01	0.00000E+00	2.72929E+04	0.00000E+00	7.10543E-14	0.00000E+00	1.20213E-09
	Middle	0.00000E+00	2.13494E+02	0.00000E+00	6.69326E+04	8.88178E-16	1.57563E-11	0.00000E+00	3.68265E-07
	Std	0.00000E+00	6.29714E+02	0.00000E+00	1.55507E+04	6.54687E-16	1.10979E-10	2.50466E-14	1.76953E-06
F24	Worst	0.00000E+00	2.17443E+00	0.00000E+00	2.33895E+01	5.57794E-02	1.05575E-01	0.00000E+00	8.54161E-02
	Average	0.00000E+00	1.03308E+00	0.00000E+00	1.74994E+01	4.35674E-03	1.07050E-02	0.00000E+00	1.49724E-02
	Best	0.00000E+00	2.71764E-01	0.00000E+00	9.89821E+00	0.00000E+00	2.44249E-15	0.00000E+00	7.86370E-11
	Middle	0.00000E+00	1.03005E+00	0.00000E+00	1.76026E+01	0.00000E+00	1.45328E-13	0.00000E+00	2.14876E-09
	Std	0.00000E+00	3.75358E-01	0.00000E+00	3.42945E+00	1.31191E-02	2.51596E-02	0.00000E+00	2.47127E-02
F25	Worst	**0.00000E+00**	7.34581E+00	4.10589E-91	3.10509E+01	1.99873E-01	3.99873E-01	1.30176E-102	1.99874E-01
	Average	**0.00000E+00**	4.64267E+00	2.65023E-92	2.67045E+01	1.39873E-01	2.19873E-01	5.20703E-104	1.52181E-01
	Best	**0.00000E+00**	1.36149E+00	1.13997E-103	2.11302E+01	9.98733E-02	9.98733E-02	1.06214E-147	9.98733E-02
	Middle	**0.00000E+00**	5.17044E+00	1.98384E-97	2.69985E+01	9.98734E-02	1.99873E-01	6.37662E-138	1.99873E-01
	Std	**0.00000E+00**	2.01386E+00	9.17092E-92	2.50903E+00	5.00000E-02	6.45497E-02	2.60351E-103	5.06855E-02
F26	Worst	0.00000E+00	2.88622E+03	0.00000E+00	9.42621E+04	1.19787E+01	1.11488E+02	3.12164E-02	2.09305E+01
	Average	0.00000E+00	1.01221E+03	0.00000E+00	6.66230E+04	2.23974E+00	1.38272E+01	1.85087E-03	5.81466E+00
	Best	0.00000E+00	1.84591E+02	0.00000E+00	3.22390E+04	0.00000E+00	6.97355E-10	0.00000E+00	5.10254E-05
	Middle	0.00000E+00	6.93962E+02	0.00000E+00	7.04475E+04	6.82121E-13	6.41523E+00	0.00000E+00	3.78067E+00
	Std	0.00000E+00	8.82931E+02	0.00000E+00	1.57466E+04	3.81545E+00	2.25146E+01	6.53930E-03	5.73202E+00
F27	Worst	8.88178E-16	2.06557E+01	8.88178E-16	8.88178E-16	2.00000E+01	2.00000E+01	8.88178E-16	2.00000E+01
	Average	8.88178E-16	2.05531E+01	8.88178E-16	8.88178E-16	2.00000E+01	2.00000E+01	8.88178E-16	2.00000E+01
	Best	8.88178E-16	2.04702E+01	8.88178E-16	8.88178E-16	2.00000E+01	2.00000E+01	8.88178E-16	2.00000E+01
	Middle	8.88178E-16	2.05442E+01	8.88178E-16	8.88178E-16	2.00000E+01	2.00000E+01	8.88178E-16	2.00000E+01
	Std	0.00000E+00	5.14753E-02	0.00000E+00	0.00000E+00	0.00000E+00	0.00000E+00	0.00000E+00	0.00000E+00

**Table 11:** Test results of the benchmark function (F1-F9, F19-F27), the dimension is fixed to 100.

Fun	Items	CSCAHHO	SCA	HHO	AOA	SOA	STOA	AO	ChOA
F1	Worst	**0.00000E+00**	1.02081E+04	8.14455E-180	2.31983E+05	2.12019E-14	9.26916E-09	3.37538E-193	6.14379E-04
	Average	**0.00000E+00**	4.29128E+03	3.25856E-181	1.81802E+05	3.62072E-15	2.16282E-09	1.35015E-194	1.27205E-04
	Best	0.00000E+00	7.13526E+01	3.36590E-208	1.15847E+05	1.21595E-16	2.19036E-13	0.00000E+00	2.23521E-06
	Middle	**0.00000E+00**	3.58736E+03	1.84842E-193	1.85197E+05	1.59961E-15	1.17172E-09	1.13417E-290	4.10763E-05
	Std	0.00000E+00	2.73885E+03	0.00000E+00	3.33200E+04	4.80035E-15	2.62754E-09	0.00000E+00	1.70634E-04
F2	Worst	**1.37155E-270**	7.10575E+01	1.51834E-92	2.49023E+03	6.35064E-09	7.01464E-06	6.22929E-99	7.53566E-03
	Average	**5.48628E-272**	1.65754E+01	7.89754E-94	1.87547E+03	1.04095E-09	1.10682E-06	2.49879E-100	2.81942E-03
	Best	**3.96930E-301**	5.97127E-02	2.11494E-108	1.24536E+03	7.58319E-11	2.44601E-08	1.12007E-148	5.46188E-04
	Middle	**3.16825E-286**	9.93630E+00	7.66572E-99	1.88040E+03	5.62752E-10	5.40757E-07	3.75940E-143	2.12805E-03
	Std	**0.00000E+00**	2.01243E+01	3.12221E-93	3.19913E+02	1.50591E-09	1.64851E-06	1.24572E-99	1.98667E-03
F3	Worst	**0.00000E+00**	6.63123E+11	1.35146E-173	1.12275E+13	8.81009E-07	1.84458E-01	2.36411E-190	4.58102E+04
	Average	**0.00000E+00**	2.25131E+11	5.40788E-175	9.22918E+12	1.21690E-07	3.81767E-02	9.45643E-192	6.06400E+03
	Best	**0.00000E+00**	2.59067E+10	7.57634E-202	6.42800E+12	1.44939E-09	7.83951E-04	1.15853E-292	3.56977E+02
	Middle	**0.00000E+00**	1.88426E+11	7.85422E-186	9.47645E+12	3.90325E-08	1.05366E-02	1.20006E-280	2.18000E+03
	Std	0.00000E+00	1.79428E+11	0.00000E+00	1.28300E+12	2.31148E-07	5.17421E-02	0.00000E+00	9.60002E+03
F4	Worst	0.00000E+00	4.78682E+05	0.00000E+00	4.78022E+08	1.18838E-32	1.96723E-19	0.00000E+00	9.70721E-12
	Average	0.00000E+00	2.46859E+04	0.00000E+00	1.81035E+08	6.90833E-34	1.44210E-20	0.00000E+00	4.01311E-13
	Best	0.00000E+00	9.20045E-01	0.00000E+00	3.86706E+06	3.96626E-40	7.84289E-30	0.00000E+00	1.60867E-19
	Middle	0.00000E+00	9.36890E+02	0.00000E+00	1.53871E+08	1.32439E-35	2.83317E-23	0.00000E+00	1.54167E-15
	Std	0.00000E+00	9.53068E+04	0.00000E+00	1.17319E+08	2.38529E-33	4.78225E-20	0.00000E+00	1.93942E-12
F5	Worst	**1.48790E-223**	9.12516E+01	2.94708E-92	9.77432E+01	8.35715E+01	9.11212E+01	1.22372E-99	8.78650E+01
	Average	**5.95583E-225**	8.68380E+01	1.19590E-93	9.59379E+01	5.57535E+01	5.77640E+01	4.89489E-101	5.09762E+01
	Best	**1.82929E-249**	8.07854E+01	8.53628E-105	9.34955E+01	3.03075E+00	2.70087E+00	7.16950E-153	1.14494E+01
	Middle	**4.64810E-238**	8.72213E+01	1.63446E-98	9.60337E+01	5.92339E+01	6.11584E+01	5.78473E-146	5.87443E+01
	Std	**0.00000E+00**	2.63229E+00	5.89082E-93	1.05898E+00	1.94461E+01	2.51516E+01	2.44745E-100	2.66459E+01
F6	Worst	**4.71872E-271**	5.77368E+01	5.72135E-95	7.85013E+01	2.47107E-09	1.02062E-06	1.15432E-98	2.53245E-03
	Average	**1.96232E-272**	1.04867E+01	3.23419E-96	5.78914E+01	3.79619E-10	3.25450E-07	4.61727E-100	8.51355E-04
	Best	**5.71292E-299**	2.24707E-01	2.39151E-104	4.16028E+01	3.15789E-11	1.18208E-08	5.82844E-152	1.02321E-04
	Middle	**1.53296E-286**	4.99155E+00	8.87033E-99	5.87637E+01	1.82518E-10	2.63766E-07	5.68044E-145	7.05620E-04
	Std	**0.00000E+00**	1.26364E+01	1.15181E-95	1.03804E+01	5.63466E-10	2.69772E-07	2.30863E-99	6.96482E-04
F7	Worst	**0.00000E+00**	6.41099E+05	7.76395E-177	8.94544E+06	8.28176E-13	1.44571E-06	1.52649E-195	5.26782E-02
	Average	**0.00000E+00**	1.78857E+05	3.10558E-178	7.31650E+06	1.43070E-13	9.82258E-08	6.10598E-197	7.20404E-03
	Best	**0.00000E+00**	3.51255E+04	2.31091E-212	5.95111E+06	3.41362E-16	8.15515E-10	2.24641E-296	1.25480E-04
	Middle	**0.00000E+00**	1.19660E+05	1.08554E-193	7.48162E+06	4.64796E-14	2.62055E-08	5.82020E-287	2.67699E-03
	Std	0.00000E+00	1.54123E+05	0.00000E+00	8.83573E+05	2.22346E-13	2.83978E-07	0.00000E+00	1.17242E-02
F8	Worst	0.00000E+00	2.51065E+08	0.00000E+00	1.62097E+09	1.09968E-21	3.36204E-12	0.00000E+00	6.92213E-03
	Average	0.00000E+00	7.77660E+07	0.00000E+00	1.38833E+09	6.95219E-23	3.63633E-13	0.00000E+00	4.91454E-04
	Best	0.00000E+00	9.41010E+06	0.00000E+00	1.13623E+09	1.25491E-28	1.42110E-16	0.00000E+00	1.21871E-07
	Middle	0.00000E+00	6.99841E+07	0.00000E+00	1.41096E+09	1.68253E-24	1.58678E-13	0.00000E+00	8.14454E-06
	Std	0.00000E+00	5.16957E+07	0.00000E+00	1.15909E+08	2.24253E-22	6.82187E-13	0.00000E+00	1.62838E-03
F9	Worst	0.00000E+00	5.26221E+09	0.00000E+00	7.33906E+10	1.32110E-20	4.57293E-10	0.00000E+00	2.43846E-01
	Average	0.00000E+00	2.26549E+09	0.00000E+00	5.90498E+10	8.97313E-22	2.23909E-11	0.00000E+00	1.33102E-02
	Best	0.00000E+00	2.89042E+08	0.00000E+00	4.71709E+10	1.73931E-26	6.09776E-16	0.00000E+00	1.39998E-06
	Middle	0.00000E+00	2.20125E+09	0.00000E+00	5.83125E+10	1.32505E-23	5.54644E-13	0.00000E+00	1.21213E-03
	Std	0.00000E+00	1.23604E+09	0.00000E+00	6.24409E+09	2.75028E-21	9.09184E-11	0.00000E+00	4.85037E-02
F19	Worst	1.00000E+00	1.00000E+00	1.00000E+00	1.00000E+00	1.00000E+00	1.00000E+00	1.00000E+00	1.00000E+00
	Average	1.00000E+00	1.00000E+00	**5.20000E-01**	1.00000E+00	1.00000E+00	1.00000E+00	8.80000E-01	1.00000E+00
	Best	1.00000E+00	1.00000E+00	**0.00000E+00**	1.00000E+00	1.00000E+00	1.00000E+00	0.00000E+00	1.00000E+00
	Middle	1.00000E+00	1.00000E+00	1.00000E+00	1.00000E+00	1.00000E+00	1.00000E+00	1.00000E+00	1.00000E+00
	Std	0.00000E+00	0.00000E+00	5.09902E-01	0.00000E+00	0.00000E+00	0.00000E+00	3.31662E-01	0.00000E+00
F20	Worst	0.00000E+00	3.66026E+12	0.00000E+00	2.40126E+13	7.36938E-24	8.40779E-13	0.00000E+00	1.60095E+00
	Average	0.00000E+00	1.40580E+12	0.00000E+00	1.92477E+13	4.09432E-25	6.10595E-14	0.00000E+00	6.83709E-02
	Best	0.00000E+00	2.38325E+11	0.00000E+00	1.51521E+13	2.67732E-34	8.70486E-21	0.00000E+00	3.30385E-08
	Middle	0.00000E+00	1.23637E+12	0.00000E+00	1.95227E+13	6.09097E-28	1.52719E-15	0.00000E+00	7.73073E-05
	Std	0.00000E+00	7.05281E+11	0.00000E+00	2.35742E+12	1.53110E-24	1.76242E-13	0.00000E+00	3.19747E-01
F21	Worst	9.79998E+01	2.04153E+10	1.25647E-01	1.55318E+11	9.87626E+01	9.87510E+01	**9.96115E-02**	1.05616E+02
	Average	5.48685E+01	7.96249E+09	1.98005E-02	1.36077E+11	9.85970E+01	9.84067E+01	**1.12517E-02**	9.95899E+01
	Best	**1.88550E-05**	1.63557E+09	9.58933E-05	1.16635E+11	9.71834E+01	9.71192E+01	1.51111E-04	9.86557E+01
	Middle	9.79962E+01	6.37672E+09	**9.81292E-03**	1.34825E+11	9.86776E+01	9.86380E+01	5.61523E-03	9.89446E+01
	Std	4.96385E+01	5.49036E+09	2.67336E-02	1.06582E+10	3.07351E-01	4.59829E-01	**2.00640E-02**	1.47842E+00
F22	Worst	**1.03835E-271**	4.27063E+02	4.02717E-04	1.37634E+03	3.71170E-03	6.92772E+01	2.35575E-03	4.66877E-01
	Average	**4.15343E-273**	2.06896E+02	1.61087E-05	9.42872E+02	2.90284E-04	4.86265E+00	2.38315E-04	4.50677E-02
	Best	**1.06345E-298**	7.23356E+01	1.08902E-108	6.44718E+02	4.20460E-10	1.72671E-06	4.44148E-149	1.86018E-03
	Middle	**6.14260E-287**	2.08249E+02	8.69129E-101	9.21817E+02	6.88556E-09	3.25879E-03	9.92211E-139	1.19500E-02
	Std	**0.00000E+00**	9.75186E+01	8.05435E-05	1.91706E+02	8.44947E-04	1.62269E+01	6.11530E-04	9.47156E-02
F23	Worst	0.00000E+00	1.35178E+04	0.00000E+00	2.31220E+05	1.97176E-13	1.13381E-07	0.00000E+00	1.48474E-02
	Average	0.00000E+00	4.03246E+03	0.00000E+00	1.96420E+05	4.41247E-14	1.48151E-08	0.00000E+00	1.91999E-03
	Best	0.00000E+00	6.09929E+02	0.00000E+00	1.33422E+05	1.24345E-14	8.96527E-12	0.00000E+00	5.13355E-05
	Middle	0.00000E+00	2.71593E+03	0.00000E+00	1.96223E+05	3.55271E-14	7.48198E-09	0.00000E+00	6.61436E-04
	Std	0.00000E+00	3.56647E+03	0.00000E+00	2.25186E+04	4.03142E-14	2.42617E-08	0.00000E+00	3.20751E-03
F24	Worst	0.00000E+00	4.98131E+00	0.00000E+00	5.74247E+01	3.55271E-15	3.50291E-02	0.00000E+00	7.33520E-02
	Average	0.00000E+00	2.52007E+00	0.00000E+00	5.01849E+01	4.39648E-16	3.80322E-03	0.00000E+00	1.22920E-02
	Best	0.00000E+00	1.18582E+00	0.00000E+00	3.85331E+01	0.00000E+00	2.08278E-13	0.00000E+00	1.42902E-08
	Middle	0.00000E+00	2.43371E+00	0.00000E+00	5.04639E+01	1.11022E-16	9.22473E-12	0.00000E+00	1.99762E-06
	Std	0.00000E+00	9.77177E-01	0.00000E+00	5.13961E+00	8.00260E-16	1.05473E-02	0.00000E+00	2.59819E-02
F25	Worst	**0.00000E+00**	1.68770E+01	8.40340E-89	5.12125E+01	2.99873E-01	3.99874E-01	7.42838E-02	2.99879E-01
	Average	**0.00000E+00**	8.38035E+00	4.24207E-90	4.56348E+01	1.79873E-01	2.79873E-01	5.90367E-03	2.12133E-01
	Best	**0.00000E+00**	3.42302E+00	4.91079E-104	3.98384E+01	9.98733E-02	1.99873E-01	2.69803E-149	9.98733E-02
	Middle	**0.00000E+00**	7.50043E+00	5.81253E-98	4.57860E+01	1.99873E-01	2.99873E-01	2.95674E-140	1.99874E-01
	Std	**0.00000E+00**	3.25219E+00	1.69194E-89	3.07272E+00	5.00000E-02	7.63763E-02	1.68766E-02	7.21666E-02
F26	Worst	0.00000E+00	1.13559E+04	0.00000E+00	6.73781E+00	2.86333E+01	1.02458E+02	0.00000E+00	6.36002E+01
	Average	0.00000E+00	5.49390E+03	0.00000E+00	3.83506E+00	4.82889E+00	2.15920E+01	0.00000E+00	2.59787E+01
	Best	0.00000E+00	7.99624E+02	0.00000E+00	1.07389E+00	3.41061E-13	2.44404E-09	0.00000E+00	2.33827E-01
	Middle	0.00000E+00	5.56311E+03	0.00000E+00	3.86581E+00	1.00741E+00	1.36991E+01	0.00000E+00	2.88527E+01
	Std	0.00000E+00	3.37900E+03	0.00000E+00	1.57433E+00	7.38529E+00	2.50642E+01	0.00000E+00	1.85175E+01
F27	Worst	8.88178E-16	2.07289E+01	8.88178E-16	8.88178E-16	2.00000E+01	2.00000E+01	8.88178E-16	2.00055E+01
	Average	8.88178E-16	2.06638E+01	8.88178E-16	8.88178E-16	2.00000E+01	2.00000E+01	8.88178E-16	2.00008E+01
	Best	8.88178E-16	2.05635E+01	8.88178E-16	8.88178E-16	2.00000E+01	2.00000E+01	8.88178E-16	2.00000E+01
	Middle	8.88178E-16	2.06681E+01	8.88178E-16	8.88178E-16	2.00000E+01	2.00000E+01	8.88178E-16	2.00000E+01
	Std	0.00000E+00	4.38098E-02	0.00000E+00	0.00000E+00	0.00000E+00	0.00000E+00	0.00000E+00	1.41242E-03

**Table 12:** Test results of the benchmark function (F1-F9, F19-F27), the dimension is fixed to 500.

Fun	Items	CSCAHHO	SCA	HHO	AOA	SOA	STOA	AO	ChOA
F1	Worst	**0.00000E+00**	2.40437E+05	2.99134E-181	6.79220E-01	7.16760E-08	8.73443E-04	4.14812E-191	5.07374E+01
	Average	**0.00000E+00**	1.51912E+05	1.19741E-182	6.14422E-01	8.61324E-09	1.53750E-04	1.65925E-192	1.62031E+01
	Best	**0.00000E+00**	5.35733E+04	1.24335E-206	5.62913E-01	1.03768E-10	2.86124E-06	1.90869E-302	3.04937E+00
	Middle	**0.00000E+00**	1.59169E+05	5.83889E-191	6.15113E-01	3.57989E-09	6.83515E-05	3.05929E-289	1.37764E+01
	Std	0.00000E+00	6.15304E+04	0.00000E+00	3.31270E-02	1.60574E-08	2.24508E-04	0.00000E+00	1.06181E+01
F2	Worst	**9.48577E-264**	1.84688E+03	4.29032E-95	8.71163E+00	3.66027E-06	3.53608E-03	9.71896E-96	1.35917E+01
	Average	**3.79628E-265**	8.99514E+02	2.47886E-96	8.00189E+00	1.36969E-06	6.07760E-04	3.92896E-97	8.53069E+00
	Best	**5.71775E-298**	1.96491E+02	4.33663E-104	6.58428E+00	3.30632E-07	1.13788E-05	3.88389E-149	5.39649E+00
	Middle	**1.17117E-283**	8.30818E+02	2.62587E-99	8.12062E+00	1.16631E-06	3.72651E-04	3.88313E-143	7.94572E+00
	Std	**0.00000E+00**	4.52143E+02	8.90197E-96	5.77542E-01	9.54853E-07	7.43881E-04	1.94304E-96	2.40017E+00
F3	Worst	**0.00000E+00**	6.85840E+13	2.27098E-171	1.66567E+08	4.86524E+01	4.61859E+05	1.56015E-214	1.11553E+10
	Average	**0.00000E+00**	4.28003E+13	1.57219E-172	1.50085E+08	5.02304E+00	3.50414E+04	6.24059E-216	3.86463E+09
	Best	**0.00000E+00**	1.11073E+13	3.84271E-208	1.34905E+08	1.33880E-01	1.30198E+02	5.20862E-287	1.06656E+09
	Middle	**0.00000E+00**	4.34835E+13	1.20649E-180	1.49902E+08	1.27678E+00	1.49161E+04	1.28990E-279	2.55767E+09
	Std	0.00000E+00	1.75709E+13	0.00000E+00	8.19680E+06	1.01653E+01	9.01785E+04	0.00000E+00	2.77705E+09
F4	Worst	0.00000E+00	1.06772E+08	0.00000E+00	6.65462E-04	3.09761E-20	3.08687E-11	0.00000E+00	2.38415E-02
	Average	0.00000E+00	2.55090E+07	0.00000E+00	5.23166E-04	4.01375E-21	3.08556E-12	0.00000E+00	2.34745E-03
	Best	0.00000E+00	2.94823E+06	0.00000E+00	3.71128E-04	2.43316E-24	6.35166E-19	0.00000E+00	4.76578E-05
	Middle	0.00000E+00	2.04537E+07	0.00000E+00	5.31944E-04	5.32060E-22	3.01050E-13	0.00000E+00	7.69935E-04
	Std	0.00000E+00	2.47932E+07	0.00000E+00	7.07227E-05	8.20991E-21	7.44364E-12	0.00000E+00	4.86069E-03
F5	Worst	**9.27541E-212**	9.93351E+01	2.34543E-91	1.98404E-01	9.91102E+01	9.94000E+01	9.48635E-101	9.88630E+01
	Average	**3.71016E-213**	9.89084E+01	1.07033E-92	1.70978E-01	9.79970E+01	9.84830E+01	3.89375E-102	9.68228E+01
	Best	**2.53517E-241**	9.77761E+01	4.91328E-105	1.55112E-01	9.63023E+01	9.72376E+01	1.58318E-151	9.22385E+01
	Middle	**3.04357E-231**	9.89904E+01	8.45864E-98	1.67696E-01	9.81036E+01	9.84407E+01	3.26141E-145	9.75638E+01
	Std	**0.00000E+00**	3.81169E-01	4.69312E-92	1.15862E-02	6.91499E-01	6.60971E-01	1.89551E-101	1.75380E+00
F6	Worst	**7.34341E-273**	1.99599E+02	1.57433E-96	2.70065E-03	6.37829E-07	8.52720E-05	1.73492E-104	1.54614E+00
	Average	**5.73137E-274**	6.92309E+01	1.23069E-97	3.77180E-04	1.90256E-07	2.10097E-05	8.67462E-106	7.70873E-01
	Best	**2.14289E-297**	1.80827E+01	2.14337E-104	4.56504E-35	8.64942E-09	2.62615E-06	9.10308E-148	3.58468E-01
	Middle	**5.90380E-282**	6.11798E+01	5.32244E-100	6.47806E-11	1.70453E-07	1.52956E-05	1.88824E-143	6.79229E-01
	Std	**0.00000E+00**	4.54267E+01	3.71047E-97	7.85137E-04	1.60972E-07	2.00540E-05	3.87941E-105	3.15729E-01
F7	Worst	**0.00000E+00**	6.70132E+07	1.55872E-176	1.51133E+02	1.67886E-05	1.18832E-01	1.37100E-221	1.05274E+04
	Average	**0.00000E+00**	4.07659E+07	6.23489E-178	1.37193E+02	2.06024E-06	2.97526E-02	5.48400E-223	2.72802E+03
	Best	**0.00000E+00**	9.88862E+06	5.02242E-207	1.14232E+02	9.49553E-08	8.60303E-04	3.01967E-303	5.75367E+02
	Middle	**0.00000E+00**	4.15079E+07	5.66353E-192	1.38193E+02	8.32691E-07	1.92913E-02	2.19202E-288	1.87637E+03
	Std	0.00000E+00	1.46551E+07	0.00000E+00	8.58341E+00	3.55744E-06	3.38518E-02	0.00000E+00	2.42157E+03
F8	Worst	0.00000E+00	3.35246E+09	0.00000E+00	7.15463E-03	2.41882E-08	1.23048E-03	0.00000E+00	1.68888E+04
	Average	0.00000E+00	1.85788E+09	0.00000E+00	5.77239E-03	2.34882E-09	2.37429E-04	0.00000E+00	1.96945E+03
	Best	0.00000E+00	1.16479E+09	0.00000E+00	4.92991E-03	1.77571E-12	3.61397E-06	0.00000E+00	3.98102E+01
	Middle	0.00000E+00	1.84507E+09	0.00000E+00	5.65786E-03	2.42854E-10	1.40570E-04	0.00000E+00	7.63045E+02
	Std	0.00000E+00	4.96987E+08	0.00000E+00	6.16990E-04	5.16227E-09	3.04852E-04	0.00000E+00	3.73405E+03
F9	Worst	0.00000E+00	7.70180E+11	0.00000E+00	1.80580E+00	3.30325E-06	9.78649E-01	0.00000E+00	1.33168E+06
	Average	0.00000E+00	4.00024E+11	0.00000E+00	1.24598E+00	3.02672E-07	1.19891E-01	0.00000E+00	3.50577E+05
	Best	0.00000E+00	1.77684E+11	0.00000E+00	9.81899E-01	2.01213E-10	3.44969E-03	0.00000E+00	1.96998E+04
	Middle	0.00000E+00	3.73047E+11	0.00000E+00	1.20210E+00	4.93194E-08	2.94877E-02	0.00000E+00	3.23077E+05
	Std	0.00000E+00	1.45801E+11	0.00000E+00	2.13144E-01	7.47308E-07	2.40286E-01	0.00000E+00	3.29504E+05
F19	Worst	1.00000E+00	1.00000E+00	1.00000E+00	1.00000E+00	1.00000E+00	1.00000E+00	1.00000E+00	1.00000E+00
	Average	1.00000E+00	1.00000E+00	**6.00000E-01**	1.00000E+00	1.00000E+00	1.00000E+00	9.20000E-01	1.00000E+00
	Best	1.00000E+00	1.00000E+00	0.00000E+00	1.00000E+00	1.00000E+00	1.00000E+00	0.00000E+00	1.00000E+00
	Middle	1.00000E+00	1.00000E+00	1.00000E+00	1.00000E+00	1.00000E+00	1.00000E+00	1.00000E+00	1.00000E+00
	Std	0.00000E+00	0.00000E+00	5.00000E-01	0.00000E+00	0.00000E+00	0.00000E+00	2.76887E-01	0.00000E+00
F20	Worst	0.00000E+00	4.68469E+13	0.00000E+00	3.08484E-04	8.05947E+00	1.12007E+03	0.00000E+00	6.62542E+07
	Average	0.00000E+00	3.35079E+13	0.00000E+00	1.75672E-04	3.75885E-01	4.93827E+01	0.00000E+00	1.05345E+07
	Best	0.00000E+00	1.69181E+13	0.00000E+00	1.04008E-04	9.44636E-09	3.28908E-04	0.00000E+00	6.83450E+04
	Middle	0.00000E+00	3.35165E+13	0.00000E+00	1.60913E-04	1.96703E-05	7.28886E-02	0.00000E+00	4.22527E+06
	Std	0.00000E+00	7.76427E+12	0.00000E+00	4.47558E-05	1.62068E+00	2.23444E+02	0.00000E+00	1.60595E+07
F21	Worst	4.93959E+02	2.89893E+11	**7.03220E-01**	5.74618E+02	4.98685E+02	5.33213E+02	1.10699E+00	1.61930E+06
	Average	2.37101E+02	1.85807E+11	**6.84037E-02**	5.63807E+02	4.98554E+02	5.06568E+02	1.68166E-01	2.66847E+05
	Best	**8.85898E-05**	1.16349E+11	9.49827E-04	5.56384E+02	4.98362E+02	4.98648E+02	3.71946E-04	9.44046E+03
	Middle	**3.01462E-03**	1.91439E+11	1.64484E-02	5.63659E+02	4.98563E+02	5.02538E+02	3.11654E-02	7.96309E+04
	Std	2.51870E+02	4.41524E+10	**1.47393E-01**	4.91209E+00	7.81533E-02	8.17388E+00	2.90640E-01	4.15990E+05
F22	Worst	2.57835E-04	2.99579E+03	**4.82540E-88**	1.35954E+00	6.75676E-03	1.25604E+02	3.32220E-02	7.52034E+01
	Average	1.46815E-05	1.43525E+03	**1.93025E-89**	1.14006E+00	1.17888E-03	6.06766E+00	2.89853E-03	3.54802E+01
	Best	**2.25644E-298**	6.28309E+02	2.01295E-106	9.55572E-01	1.58095E-06	1.54872E-03	1.85640E-147	1.14061E+01
	Middle	**4.92106E-284**	1.36471E+03	5.02887E-100	1.14720E+00	4.58520E-04	3.52818E-01	7.20209E-86	3.32191E+01
	Std	5.51570E-05	4.84939E+02	**9.65078E-89**	1.05852E-01	1.75030E-03	2.49595E+01	7.04063E-03	1.49868E+01
F23	Worst	0.00000E+00	4.31914E+05	0.00000E+00	6.56769E+00	3.64000E-07	3.41103E-01	6.20659E-11	3.92818E+01
	Average	0.00000E+00	1.59374E+05	0.00000E+00	5.97850E+00	8.89443E-08	1.43110E-01	2.48292E-12	2.17842E+01
	Best	0.00000E+00	2.05541E+04	0.00000E+00	5.06273E+00	7.09527E-09	1.75316E-05	0.00000E+00	1.26240E+01
	Middle	0.00000E+00	1.59376E+05	0.00000E+00	6.00804E+00	5.23145E-08	1.52428E-01	0.00000E+00	2.05090E+01
	Std	0.00000E+00	9.83324E+04	0.00000E+00	4.40514E-01	1.03256E-07	1.37027E-01	1.24131E-11	6.26897E+00
F24	Worst	0.00000E+00	6.82256E+01	0.00000E+00	3.63544E-03	6.10811E-02	1.34362E-01	0.00000E+00	4.07299E-01
	Average	0.00000E+00	4.12820E+01	0.00000E+00	2.75333E-03	2.44324E-03	1.38223E-02	0.00000E+00	8.75385E-02
	Best	0.00000E+00	1.46894E+01	0.00000E+00	2.31752E-03	1.46783E-12	4.42090E-09	0.00000E+00	1.16863E-02
	Middle	0.00000E+00	4.52911E+01	0.00000E+00	2.76431E-03	1.84381E-11	4.13693E-07	0.00000E+00	4.54941E-02
	Std	0.00000E+00	1.62804E+01	0.00000E+00	2.98508E-04	1.22162E-02	3.53064E-02	0.00000E+00	1.15876E-01
F25	Worst	**0.00000E+00**	5.71064E+01	1.39571E-92	3.99874E-01	3.99873E-01	5.99873E-01	1.00022E-01	1.80713E+00
	Average	**0.00000E+00**	4.08983E+01	9.40118E-94	2.47874E-01	2.71873E-01	4.95873E-01	6.87201E-03	1.31691E+00
	Best	**0.00000E+00**	2.53315E+01	5.83499E-103	9.98734E-02	1.99873E-01	2.99873E-01	3.72548E-146	9.43633E-01
	Middle	**0.00000E+00**	4.26649E+01	2.28048E-97	1.99874E-01	2.99873E-01	4.99873E-01	1.40463E-139	1.38154E+00
	Std	**0.00000E+00**	7.46381E+00	3.19682E-93	8.22596E-02	6.13732E-02	7.89515E-02	2.19623E-02	2.31070E-01
F26	Worst	0.00000E+00	3.18998E+05	0.00000E+00	1.30265E+02	3.33846E+01	2.59874E+02	5.18183E-01	9.06286E+02
	Average	0.00000E+00	1.63067E+05	0.00000E+00	1.15204E+02	1.02408E+01	7.48457E+01	2.60618E-02	5.77674E+02
	Best	0.00000E+00	5.36060E+04	0.00000E+00	1.03004E+02	2.67907E-07	1.48675E+01	0.00000E+00	3.52104E+02
	Middle	0.00000E+00	1.39598E+05	0.00000E+00	1.12834E+02	8.86754E+00	5.16741E+01	0.00000E+00	5.82298E+02
	Std	0.00000E+00	7.49399E+04	0.00000E+00	7.75523E+00	9.98396E+00	6.27326E+01	1.04397E-01	1.28786E+02
F27	Worst	8.88178E-16	2.09376E+01	8.88178E-16	2.14114E-01	2.00000E+01	2.00000E+01	8.88178E-16	2.01002E+01
	Average	8.88178E-16	2.08761E+01	8.88178E-16	1.99075E-01	2.00000E+01	2.00000E+01	8.88178E-16	2.00886E+01
	Best	8.88178E-16	2.08289E+01	8.88178E-16	1.87899E-01	2.00000E+01	2.00000E+01	8.88178E-16	2.00668E+01
	Middle	8.88178E-16	2.08769E+01	8.88178E-16	1.98963E-01	2.00000E+01	2.00000E+01	8.88178E-16	2.00912E+01
	Std	0.00000E+00	2.70657E-02	0.00000E+00	6.55082E-03	0.00000E+00	1.31748E-10	0.00000E+00	8.75984E-03

From Tables 9 to 11, we can find that the proposed CSCAHHO algorithm could perform optimization better than others at most times. However, Some of other algorithms such as the STOA, AOA, HHO and so on would also achieve the same condition for some easy problems.

### 4.7 Friedman statistical test

Friedman statistical test is usually introduced to verify whether the result of the proposed algorithm is better or in the same level with those obtained by other algorithms. In this section, simulation experiments would be carried out for 30 times, and Friedman statistical test would be checked, results were shown in Table 13.

**Table 13:** The results of the Friedman statistical test on 27 functions.

F	CSCAHHO vs SCA	CSCAHHO vs HHO	CSCAHHO vs AOA	CSCAHHO vs SOA	CSCAHHO vs STOA	CSCAHHO vs AO	CSCAHHO vs ChOA
F1	1.22903E-05	1.22903E-05	1.22903E-05	1.22903E-05	1.22903E-05	1.22903E-05	1.22903E-05
F2	1.22903E-05	1.22903E-05	1.22903E-05	1.22903E-05	1.22903E-05	1.22903E-05	1.22903E-05
F3	1.22903E-05	1.22903E-05	1.22903E-05	1.22903E-05	1.22903E-05	1.22903E-05	1.22903E-05
F4	1.22903E-05	NaN	2.70159E-05	1.22903E-05	1.22903E-05	NaN	1.22903E-05
F5	1.22903E-05	1.22903E-05	1.22903E-05	1.22903E-05	1.22903E-05	1.22903E-05	1.22903E-05
F6	1.22903E-05	1.22903E-05	1.22903E-05	1.22903E-05	1.22903E-05	1.22903E-05	1.22903E-05
F7	1.22903E-05	1.22903E-05	1.22903E-05	1.22903E-05	1.22903E-05	1.22903E-05	1.22903E-05
F8	1.22903E-05	NaN	1.22903E-05	1.22903E-05	1.22903E-05	NaN	1.22903E-05
F9	1.22903E-05	NaN	1.22903E-05	1.22903E-05	1.22903E-05	NaN	1.22903E-05
F10	1.22903E-05	1.22903E-05	2.72650E-06	1.22903E-05	1.22903E-05	1.22903E-05	1.22903E-05
F11	1.22903E-05	**1.74210E-01**	1.22903E-05	1.22903E-05	1.22903E-05	1.22903E-05	1.22903E-05
F12	1.22903E-05	1.22903E-05	NaN	1.22903E-05	1.22903E-05	1.22903E-05	1.22903E-05
F13	NaN	NaN	NaN	**2.50000E-01**	**1.25000E-01**	NaN	2.70159E-05
F14	NaN	NaN	NaN	**2.50000E-01**	1.56250E-02	NaN	2.93053E-04
F15	1.22903E-05	1.22903E-05	NaN	1.22903E-05	1.22903E-05	1.22903E-05	1.22903E-05
F16	1.22903E-05	1.22903E-05	NaN	1.22903E-05	1.22903E-05	1.22903E-05	1.78597E-05
F17	1.22903E-05	1.22903E-05	NaN	1.22903E-05	1.22903E-05	1.22903E-05	1.22903E-05
F18	NaN	NaN	NaN	NaN	NaN	**5.00000E-01**	NaN
F19	1.22903E-05	NaN	1.22903E-05	1.22903E-05	1.22903E-05	**1.25000E-01**	1.22903E-05
F20	1.22903E-05	NaN	1.22903E-05	1.22903E-05	1.22903E-05	NaN	1.22903E-05
F21	1.22903E-05	7.22447E-05	1.22903E-05	1.22903E-05	1.22903E-05	1.57051E-05	1.22903E-05
F22	1.22903E-05	1.22903E-05	1.22903E-05	1.22903E-05	1.22903E-05	1.22903E-05	1.22903E-05
F23	1.22903E-05	NaN	1.22903E-05	NaN	9.65591E-06	NaN	1.22903E-05
F24	1.22903E-05	NaN	1.22903E-05	**5.00000E-01**	3.90625E-03	NaN	1.93023E-04
F25	1.22903E-05	1.22903E-05	1.22903E-05	1.22903E-05	1.22903E-05	1.22903E-05	1.22903E-05
F26	1.22903E-05	NaN	1.22903E-05	9.76563E-04	1.81974E-05	**5.00000E-01**	1.22903E-05
F27	1.22903E-05	NaN	NaN	1.82930E-06	3.13266E-06	NaN	8.94473E-07

Results in Table 13 showed that the proposed CSCAHHO algorithm would perform significantly better than other algorithms almost all the times, we can further draw the conclusion that the proposed CSCAHHO algorithm would perform quite better in optimizing traditional benchmark functions, either they are unimodal or multimodal, scalable or in fixed dimensionality.

### 4.8 Experiments on the CEC2014 benchmark function

In order to verify the better performance of the proposed CSCAHHO algorithm, we would further carry on some simulation experiments on the CEC 2014 competitive problems. The results were shown in Table 14.

**Table 14:** Results of CEC2014 benchmark function.

Fun	Items	CSCAHHO	SCA	HHO	AOA	SOA	STOA	AO	ChOA
F28	Worst	1.71395E+07	1.00397E+09	7.76482E+08	2.52401E+07	**3.97845E+06**	1.37396E+08	1.47355E+07	3.10201E+07
	Average	5.38223E+06	3.44838E+08	2.47627E+08	1.39393E+07	**1.65883E+06**	8.00162E+07	6.84233E+06	1.60359E+07
	Best	**2.32066E+05**	7.46727E+07	3.12265E+07	2.21431E+06	2.40373E+05	4.44934E+07	1.21758E+06	6.31654E+06
	Middle	**2.66540E+06**	3.27620E+08	2.13865E+08	1.41139E+07	2.67865E+06	7.51751E+07	6.89097E+06	1.48162E+07
	Std	5.55244E+06	2.23214E+08	1.78415E+08	6.46799E+06	**1.00972E+06**	2.62377E+07	3.71770E+06	7.24014E+06
F29	Worst	3.44296E+08	3.65860E+10	3.04845E+10	1.18740E+10	9.17028E+08	2.69878E+10	**2.90832E+08**	3.18518E+09
	Average	**2.82384E+07**	2.06994E+10	1.73187E+10	5.43151E+09	2.34890E+08	1.96662E+10	6.82467E+07	1.86521E+09
	Best	**1.67409E+05**	3.80946E+09	8.23005E+09	8.58040E+08	2.63085E+06	1.15715E+10	1.04229E+06	6.89904E+08
	Middle	**1.50776E+06**	2.28962E+10	1.72383E+10	4.71652E+09	8.28047E+07	1.97992E+10	4.36106E+07	1.91490E+09
	Std	8.98196E+07	8.91704E+09	5.77974E+09	3.44853E+09	2.44926E+08	3.64878E+09	**7.26685E+07**	5.59772E+08
F30	Worst	**1.60042E+04**	3.36871E+07	1.30498E+05	4.03200E+04	2.47130E+04	1.25773E+05	5.18701E+04	5.51314E+04
	Average	**8.73916E+03**	3.79598E+06	6.49476E+04	1.86955E+04	1.28571E+04	9.44898E+04	2.61113E+04	3.87573E+04
	Best	**1.54721E+03**	2.45063E+05	9.62049E+03	4.73592E+03	2.43883E+03	4.33530E+04	8.40726E+03	2.36604E+04
	Middle	**8.96109E+03**	1.05666E+06	6.61886E+04	1.87909E+04	1.30421E+04	9.41536E+04	2.40381E+04	3.98399E+04
	Std	**3.30240E+03**	7.36018E+06	3.36162E+04	7.84440E+03	5.08533E+03	1.48392E+04	1.14990E+04	8.63564E+03
F31	Worst	5.94666E+02	4.77510E+03	5.63729E+03	2.37046E+03	**5.03419E+02**	3.22275E+03	5.08691E+02	8.28361E+02
	Average	**4.46989E+02**	2.39017E+03	3.02429E+03	1.01421E+03	4.49211E+02	1.93420E+03	4.47233E+02	5.32517E+02
	Best	**4.00497E+02**	8.45140E+02	1.09346E+03	5.14679E+02	4.07044E+02	9.39657E+02	4.06799E+02	4.66722E+02
	Middle	**4.18530E+02**	1.89902E+03	3.01566E+03	8.80928E+02	4.55203E+02	1.86272E+03	4.53659E+02	5.09717E+02
	Std	5.37654E+01	1.21399E+03	1.33492E+03	4.76761E+02	2.76141E+01	6.53390E+02	**2.75505E+01**	7.00998E+01
F32	Worst	**5.20298E+02**	5.21130E+02	5.21367E+02	5.20167E+02	5.20573E+02	5.20518E+02	5.20636E+02	5.20687E+02
	Average	5.20118E+02	5.20936E+02	5.21000E+02	**5.20113E+02**	5.20368E+02	5.20414E+02	5.20307E+02	5.20475E+02
	Best	**5.20020E+02**	5.20639E+02	5.20477E+02	5.20020E+02	5.20237E+02	5.20296E+02	5.20141E+02	5.20325E+02
	Middle	**5.20109E+02**	5.20969E+02	5.21044E+02	5.20109E+02	5.20348E+02	5.20415E+02	5.20291E+02	5.20493E+02
	Std	7.22215E-02	1.07008E-01	2.16503E-01	3.68835E-02	9.50640E-02	**5.57774E-02**	1.24123E-01	9.43856E-02
F33	Worst	6.10647E+02	6.15210E+02	6.16117E+02	6.12800E+02	**6.08288E+02**	6.11754E+02	6.09576E+02	6.10912E+02
	Average	**6.07198E+02**	6.13552E+02	6.14178E+02	6.10529E+02	6.05987E+02	6.10619E+02	6.07569E+02	6.09157E+02
	Best	**6.02657E+02**	6.10886E+02	6.11608E+02	6.08822E+02	6.02743E+02	6.09182E+02	6.05774E+02	6.07660E+02
	Middle	**6.07198E+02**	6.13725E+02	6.14320E+02	6.10441E+02	6.06590E+02	6.10635E+02	6.07558E+02	6.09259E+02
	Std	1.71466E+00	1.09957E+00	1.12173E+00	1.14507E+00	1.55597E+00	**5.38135E-01**	9.61218E-01	8.60276E-01
F34	Worst	**7.02325E+02**	9.97454E+02	9.92970E+02	8.03906E+02	7.16404E+02	1.01711E+03	7.09207E+02	7.48570E+02
	Average	**7.00783E+02**	8.89678E+02	8.97904E+02	7.68204E+02	7.03948E+02	9.16765E+02	7.03079E+02	7.30271E+02
	Best	**7.00128E+02**	7.81742E+02	7.99321E+02	7.20753E+02	7.00709E+02	8.09168E+02	7.00922E+02	7.14098E+02
	Middle	**7.00620E+02**	8.86885E+02	9.02057E+02	7.64904E+02	7.01915E+02	9.26533E+02	7.02540E+02	7.29329E+02
	Std	**5.80422E-01**	4.66637E+01	5.38474E+01	2.19576E+01	4.36831E+00	4.78222E+01	1.88972E+00	8.96285E+00
F35	Worst	**8.34015E+02**	9.46675E+02	9.49362E+02	8.40889E+02	8.49608E+02	9.77067E+02	8.36874E+02	8.78173E+02
	Average	**8.19446E+02**	9.23506E+02	9.14854E+02	8.25984E+02	8.22954E+02	9.35172E+02	8.25950E+02	8.64611E+02
	Best	**8.06068E+02**	8.89380E+02	8.64400E+02	8.16754E+02	8.06920E+02	8.88700E+02	8.08087E+02	8.48297E+02
	Middle	**8.18986E+02**	9.22485E+02	9.16253E+02	8.25910E+02	8.20315E+02	9.41756E+02	8.28030E+02	8.64091E+02
	Std	**5.17051E+00**	1.61915E+01	1.85894E+01	6.60338E+00	1.06296E+01	2.21987E+01	7.81611E+00	8.31064E+00
F36	Worst	**9.35819E+02**	1.06561E+03	1.06795E+03	9.44514E+02	9.46680E+02	1.07144E+03	9.48232E+02	9.79864E+02
	Average	**9.23971E+02**	1.03205E+03	1.02757E+03	9.27575E+02	9.24270E+02	1.03706E+03	9.27004E+02	9.69128E+02
	Best	**9.10754E+02**	9.90492E+02	9.87251E+02	9.14090E+02	9.10770E+02	9.95189E+02	9.15093E+02	9.42058E+02
	Middle	9.22900E+02	1.03282E+03	1.02858E+03	9.25877E+02	**9.22421E+02**	1.04784E+03	9.25285E+02	9.70648E+02
	Std	**7.24404E+00**	2.36613E+01	2.09324E+01	9.26159E+00	8.62532E+00	2.57141E+01	7.57981E+00	8.03071E+00
F37	Worst	**2.21629E+03**	3.58236E+03	4.11100E+03	2.47590E+03	2.31581E+03	2.85176E+03	2.23217E+03	2.36862E+03
	Average	**1.58506E+03**	3.22274E+03	3.54606E+03	1.98275E+03	1.73747E+03	2.65743E+03	1.77558E+03	2.12900E+03
	Best	**1.01967E+03**	2.49008E+03	2.97298E+03	1.60011E+03	1.34442E+03	2.47117E+03	1.14454E+03	1.70527E+03
	Middle	**1.54461E+03**	3.26643E+03	3.56987E+03	2.00810E+03	1.72681E+03	2.61969E+03	1.80718E+03	2.15549E+03
	Std	3.27707E+02	2.76941E+02	3.13603E+02	2.17156E+02	2.44275E+02	9.00998E+01	2.78003E+02	**1.65977E+02**
F38	Worst	2.76579E+03	4.18653E+03	4.17621E+03	**2.59018E+03**	2.79192E+03	3.17984E+03	2.43393E+03	3.07848E+03
	Average	**2.07670E+03**	3.64827E+03	3.75662E+03	2.11689E+03	2.24218E+03	3.03285E+03	2.15982E+03	2.92028E+03
	Best	**1.50239E+03**	2.90983E+03	2.99150E+03	1.58639E+03	1.85311E+03	2.70811E+03	1.80017E+03	2.56191E+03
	Middle	**2.04586E+03**	3.69418E+03	3.81851E+03	2.16208E+03	2.20899E+03	3.04775E+03	2.17945E+03	2.94438E+03
	Std	2.99919E+02	3.03082E+02	2.88375E+02	2.75067E+02	2.56584E+02	1.18526E+02	1.99317E+02	**1.15568E+02**
F39	Worst	1.20122E+03	1.20452E+03	1.20571E+03	**1.20080E+03**	1.20120E+03	1.20205E+03	1.20168E+03	1.20166E+03
	Average	1.20070E+03	1.20306E+03	1.20319E+03	**1.20040E+03**	1.20074E+03	1.20145E+03	1.20068E+03	1.20126E+03
	Best	**1.20012E+03**	1.20193E+03	1.20172E+03	1.20013E+03	1.20047E+03	1.20100E+03	1.20014E+03	1.20082E+03
	Middle	**1.20060E+03**	1.20295E+03	1.20302E+03	1.20077E+03	1.20068E+03	1.20147E+03	1.20064E+03	1.20124E+03
	Std	2.81867E-01	6.21139E-01	1.02362E+00	**1.73571E-01**	2.04124E-01	2.38900E-01	3.57123E-01	2.55431E-01
F40	Worst	**1.30109E+03**	1.30679E+03	1.30594E+03	1.30377E+03	1.30179E+03	1.30610E+03	1.30127E+03	1.30147E+03
	Average	**1.30061E+03**	1.30531E+03	1.30464E+03	1.30237E+03	1.30069E+03	1.30504E+03	1.30076E+03	1.30114E+03
	Best	**1.30030E+03**	1.30353E+03	1.30241E+03	1.30055E+03	1.30036E+03	1.30411E+03	1.30027E+03	1.30070E+03
	Middle	**1.30056E+03**	1.30536E+03	1.30481E+03	1.30259E+03	1.30057E+03	1.30508E+03	1.30073E+03	1.30112E+03
	Std	**2.02335E-01**	7.51679E-01	9.38971E-01	9.37865E-01	2.31361E-01	5.25004E-01	2.78804E-01	1.84274E-01
F41	Worst	**1.40082E+03**	1.49733E+03	1.49890E+03	1.45195E+03	1.40089E+03	1.46228E+03	1.40125E+03	1.40589E+03
	Average	**1.40034E+03**	1.45303E+03	1.45621E+03	1.42695E+03	1.40061E+03	1.44514E+03	1.40061E+03	1.40387E+03
	Best	**1.40013E+03**	1.42186E+03	1.40476E+03	1.41474E+03	1.40024E+03	1.42604E+03	1.40028E+03	1.40225E+03
	Middle	**1.40027E+03**	1.45689E+03	1.45532E+03	1.42677E+03	1.40070E+03	1.44847E+03	1.40043E+03	1.40372E+03
	Std	2.20518E-01	1.73751E+01	2.28120E+01	7.68058E+00	**1.92489E-01**	8.93192E+00	3.16728E-01	1.02896E+00
F42	Worst	**1.54749E+03**	3.64950E+07	6.86478E+07	1.91124E+05	2.31662E+03	1.46146E+07	1.56578E+03	2.73055E+03
	Average	**1.51210E+03**	6.50695E+06	8.82389E+06	3.20466E+04	1.54166E+03	4.75174E+06	1.52538E+03	1.83669E+03
	Best	**1.50246E+03**	2.39403E+04	3.01784E+05	1.61596E+03	1.50269E+03	2.85490E+05	1.50457E+03	1.54221E+03
	Middle	1.50878E+03	3.03390E+06	3.91231E+06	1.42801E+04	**1.50661E+03**	2.61416E+06	1.52064E+03	1.73422E+03
	Std	**1.07749E+01**	8.88154E+06	1.49600E+07	5.19201E+04	1.61648E+02	4.07788E+06	1.56372E+01	2.89134E+02
F43	Worst	**1.60401E+03**	1.60475E+03	1.60492E+03	1.60451E+03	1.60403E+03	1.60438E+03	1.60410E+03	1.60413E+03
	Average	**1.60350E+03**	1.60461E+03	1.60462E+03	1.60406E+03	1.60344E+03	1.60420E+03	1.60374E+03	1.60396E+03
	Best	1.60319E+03	1.60430E+03	1.60418E+03	1.60335E+03	**1.60256E+03**	1.60371E+03	1.60310E+03	1.60364E+03
	Middle	**1.60351E+03**	1.60463E+03	1.60461E+03	1.60413E+03	1.60358E+03	1.60424E+03	1.60378E+03	1.60398E+03
	Std	2.34880E-01	**1.16476E-01**	1.64139E-01	3.53857E-01	3.25600E-01	1.39967E-01	2.44810E-01	1.15235E-01
F44	Worst	1.04074E+05	2.76104E+08	1.29562E+08	1.13648E+06	**1.01009E+05**	4.20837E+06	1.13781E+06	9.38313E+05
	Average	**4.84504E+04**	5.45118E+07	3.10869E+07	3.06236E+05	6.00203E+04	1.40338E+06	2.20658E+05	1.51847E+05
	Best	5.37624E+03	1.65967E+06	3.88541E+05	1.03469E+04	**2.97890E+03**	1.47664E+05	4.52819E+03	3.00097E+04
	Middle	**4.78964E+04**	3.69329E+07	2.21564E+07	1.41523E+05	9.45232E+04	1.31348E+06	9.47604E+04	6.88937E+04
	Std	**3.14320E+04**	6.62971E+07	3.14514E+07	3.74927E+05	4.27295E+04	1.03341E+06	3.34772E+05	2.16465E+05
F45	Worst	**1.65082E+04**	3.36834E+09	2.12479E+09	2.25032E+04	4.58951E+04	3.30200E+07	2.88003E+04	4.33572E+06
	Average	6.98690E+03	9.02945E+08	6.60605E+08	**5.45337E+03**	1.87661E+04	2.73612E+07	1.06934E+04	9.04451E+05
	Best	**2.12208E+03**	4.91978E+07	7.97037E+06	2.16065E+03	2.54401E+03	6.13499E+06	2.49945E+03	8.28803E+03
	Middle	5.99474E+03	3.63955E+08	3.82318E+08	**3.11818E+03**	1.84219E+04	3.29571E+07	6.94022E+03	4.40749E+05
	Std	**4.32896E+03**	1.05319E+09	6.73847E+08	4.36934E+03	1.17175E+04	9.45922E+06	8.32015E+03	1.15102E+06
F46	Worst	**1.91096E+03**	2.41801E+03	2.33048E+03	2.05631E+03	1.91222E+03	2.14709E+03	1.91286E+03	1.91481E+03
	Average	**1.90489E+03**	2.10817E+03	2.10542E+03	1.96715E+03	1.90508E+03	2.03726E+03	1.90666E+03	1.91095E+03
	Best	**1.90233E+03**	1.93481E+03	1.91624E+03	1.90993E+03	1.90286E+03	1.94387E+03	1.90279E+03	1.90557E+03
	Middle	**1.90394E+03**	2.06215E+03	2.11733E+03	1.96564E+03	1.90422E+03	2.03473E+03	1.90644E+03	1.91097E+03
	Std	**2.44233E+00**	1.45419E+02	1.19991E+02	3.83728E+01	2.36184E+00	5.02854E+01	2.83720E+00	1.99737E+00
F47	Worst	1.11057E+04	1.49231E+08	8.74375E+07	2.89588E+05	**7.29709E+03**	3.27904E+05	3.31013E+04	6.75535E+04
	Average	**3.69670E+03**	1.49911E+07	7.80932E+06	1.11095E+05	3.81426E+03	1.92583E+05	7.96569E+03	1.33546E+04
	Best	**2.04789E+03**	1.58206E+05	1.19319E+04	2.54234E+03	2.20114E+03	1.46280E+04	2.21525E+03	2.68609E+03
	Middle	**2.77051E+03**	3.93895E+06	9.22458E+05	1.06168E+05	3.33606E+03	2.79232E+05	5.47368E+03	1.03680E+04
	Std	2.31097E+03	3.19881E+07	1.85855E+07	8.00613E+04	**1.44936E+03**	1.21890E+05	7.18430E+03	1.28428E+04
F48	Worst	**2.10775E+04**	2.85404E+06	1.15374E+07	6.87509E+04	2.22772E+04	1.10709E+05	2.70083E+04	2.75095E+04
	Average	**8.59642E+03**	7.11948E+05	1.35740E+06	1.29133E+04	1.09403E+04	2.43330E+04	1.37649E+04	1.22925E+04
	Best	**2.23299E+03**	1.38275E+04	3.63796E+03	4.49189E+03	2.82294E+03	7.64732E+03	3.95025E+03	4.95752E+03
	Middle	8.49492E+03	2.46278E+05	2.41190E+05	9.92638E+03	**5.57006E+03**	2.28247E+04	1.18570E+04	1.24914E+04
	Std	**3.88570E+03**	8.35675E+05	2.53897E+06	1.26167E+04	8.03168E+03	1.93786E+04	7.17472E+03	3.95181E+03
F49	Worst	**2.46789E+03**	3.19440E+03	9.25413E+03	2.84945E+03	2.48029E+03	2.51669E+03	2.49149E+03	2.47464E+03
	Average	2.31030E+03	2.83309E+03	3.42076E+03	2.48197E+03	2.24718E+03	2.40806E+03	**2.26591E+03**	2.35909E+03
	Best	**2.22309E+03**	2.47805E+03	2.72270E+03	2.23413E+03	2.22788E+03	2.24778E+03	2.22658E+03	2.28400E+03
	Middle	2.29735E+03	2.82132E+03	3.06134E+03	2.46918E+03	**2.23222E+03**	2.41491E+03	2.24933E+03	2.35083E+03
	Std	6.65655E+01	2.10268E+02	1.27784E+03	1.14569E+02	4.87620E+01	5.54631E+01	5.70411E+01	**4.77170E+01**
F50	Worst	**2.73017E+03**	3.33983E+03	3.38393E+03	2.94094E+03	2.79112E+03	2.92839E+03	2.74116E+03	2.77206E+03
	Average	**2.69439E+03**	2.96632E+03	3.03824E+03	2.81195E+03	2.70887E+03	2.83335E+03	2.69947E+03	2.74033E+03
	Best	**2.59226E+03**	2.79572E+03	2.77091E+03	2.72876E+03	2.59380E+03	2.72487E+03	2.59460E+03	2.71386E+03
	Middle	**2.71095E+03**	2.97197E+03	3.04578E+03	2.80727E+03	2.71182E+03	2.83460E+03	2.72209E+03	2.73428E+03
	Std	3.95909E+01	1.42139E+02	1.81459E+02	5.02608E+01	3.13678E+01	4.11359E+01	4.82162E+01	**1.72839E+01**
F51	Worst	**2.60118E+03**	2.67737E+03	2.68892E+03	2.63147E+03	2.60189E+03	2.66885E+03	2.60146E+03	2.60584E+03
	Average	2.55576E+03	2.64339E+03	2.64681E+03	2.57367E+03	2.57147E+03	2.64407E+03	**2.54330E+03**	2.60273E+03
	Best	**2.51645E+03**	2.60927E+03	2.59434E+03	2.51819E+03	2.52177E+03	2.61753E+03	2.51943E+03	2.59136E+03
	Middle	2.54642E+03	2.64660E+03	2.64410E+03	2.58068E+03	2.60059E+03	2.64812E+03	**2.53544E+03**	2.60345E+03
	Std	3.14872E+01	1.66975E+01	2.14595E+01	3.87398E+01	3.43700E+01	**1.53148E+01**	2.80521E+01	2.96998E+00
F52	Worst	**2.70882E+03**	2.80267E+03	2.79992E+03	2.71358E+03	2.70940E+03	2.71556E+03	2.70919E+03	2.70887E+03
	Average	**2.70080E+03**	2.75368E+03	2.74317E+03	2.70739E+03	2.70338E+03	2.70971E+03	2.70281E+03	2.70451E+03
	Best	**2.65871E+03**	2.72695E+03	2.70505E+03	2.68710E+03	2.70070E+03	2.70694E+03	2.68901E+03	2.68950E+03
	Middle	**2.70226E+03**	2.75136E+03	2.73944E+03	2.70762E+03	2.70296E+03	2.70836E+03	2.70341E+03	2.70518E+03
	Std	9.41652E+00	2.08363E+01	2.39340E+01	5.24458E+00	**1.68298E+00**	2.67414E+00	3.58729E+00	3.41246E+00
F53	Worst	**2.70086E+03**	2.79613E+03	2.94490E+03	2.77089E+03	2.70091E+03	2.70615E+03	2.70105E+03	2.70172E+03
	Average	**2.70050E+03**	2.71104E+03	2.72045E+03	2.70772E+03	2.70050E+03	2.70497E+03	2.70065E+03	2.70120E+03
	Best	**2.70027E+03**	2.70280E+03	2.70303E+03	2.70076E+03	2.70028E+03	2.70349E+03	2.70028E+03	2.70087E+03
	Middle	**2.70052E+03**	2.70627E+03	2.70519E+03	2.70357E+03	2.70053E+03	2.70510E+03	2.70064E+03	2.70121E+03
	Std	**1.51838E-01**	1.83927E+01	5.23986E+01	1.51214E+01	1.52366E-01	6.28816E-01	2.07515E-01	2.21434E-01
F54	Worst	3.17129E+03	3.47763E+03	3.45132E+03	3.32912E+03	3.13045E+03	3.22534E+03	**3.11756E+03**	3.22817E+03
	Average	3.10235E+03	3.35850E+03	3.32693E+03	3.19428E+03	3.07166E+03	3.18551E+03	**2.96890E+03**	3.11470E+03
	Best	**2.70612E+03**	3.24885E+03	3.07296E+03	3.10900E+03	2.70645E+03	3.13707E+03	2.71023E+03	2.90536E+03
	Middle	**3.11028E+03**	3.38389E+03	3.33701E+03	3.20453E+03	3.11243E+03	3.18221E+03	3.11072E+03	3.13200E+03
	Std	8.47912E+01	6.31063E+01	8.31868E+01	**6.28709E+01**	1.10130E+02	2.66668E+01	1.94389E+02	9.68926E+01
F55	Worst	3.36250E+03	3.93622E+03	4.48065E+03	3.58133E+03	3.44604E+03	**3.20775E+03**	3.23240E+03	3.23418E+03
	Average	3.19880E+03	3.62421E+03	3.80258E+03	3.38999E+03	**3.13994E+03**	3.17517E+03	3.18798E+03	3.16907E+03
	Best	**3.13790E+03**	3.44299E+03	3.33291E+03	3.23599E+03	3.13635E+03	3.15130E+03	3.15258E+03	3.15356E+03
	Middle	3.17317E+03	3.62011E+03	3.74090E+03	3.36669E+03	**3.13995E+03**	3.16726E+03	3.18642E+03	3.16204E+03
	Std	6.29728E+01	1.11799E+02	2.66380E+02	9.25815E+01	**2.09887E+00**	1.68864E+01	2.06167E+01	1.93694E+01
F56	Worst	8.58935E+03	2.88950E+06	6.83841E+07	4.81929E+06	**7.17088E+03**	1.12581E+04	9.04749E+03	5.34504E+04
	Average	4.50876E+03	1.03836E+06	1.34151E+07	2.74393E+05	4.15325E+03	5.95076E+03	**3.96821E+03**	1.69907E+04
	Best	**3.23333E+03**	3.81699E+04	7.19198E+03	3.34522E+03	3.30215E+03	3.91773E+03	3.23811E+03	4.41637E+03
	Middle	**3.86800E+03**	9.68478E+05	4.17605E+06	4.90178E+03	3.89174E+03	5.43681E+03	3.90512E+03	1.38200E+04
	Std	1.56807E+03	8.65542E+05	1.67501E+07	9.79215E+05	**9.23938E+02**	1.99878E+03	1.37827E+03	1.34243E+04
F57	Worst	5.92066E+03	1.53202E+07	1.80968E+06	5.53094E+04	**3.98844E+03**	6.74680E+03	1.26728E+04	6.04027E+03
	Average	4.24044E+03	1.53185E+06	3.88281E+05	1.57983E+04	**3.74397E+03**	4.66886E+03	5.44903E+03	4.42968E+03
	Best	**3.43153E+03**	1.62303E+04	2.11518E+04	5.72601E+03	3.49927E+03	4.13785E+03	3.80904E+03	3.78853E+03
	Middle	4.05779E+03	3.81863E+05	2.10505E+05	1.02528E+04	**3.73344E+03**	4.58265E+03	4.57118E+03	4.39393E+03
	Std	6.60457E+02	3.16127E+06	5.17571E+05	1.31928E+04	**1.28873E+02**	4.95553E+02	2.08844E+03	4.93755E+02

There are totally 150 best values in results of simulation experiments on CEC2014 test functions as showed in Table 14. 99 of them are achieved by the proposed CSCAHHO algorithm accounting for 66%.

## 5 Solving engineering design problems

An apparent conclusion could be drawn that the proposed CSCAHHO algorithm could perform well on benchmark functions, either unimodal or multimodal, scalable or non-scalable, benchmark or CEC competitive. The overall results are so promising that we could not resist the temptation applying in solving real engineering problems. Therefore, we would further carry on some simulation experiments on classical real engineering problems, which are also used world-wide in testing the capability of optimization algorithms.

In this section, the results would also be compiled to those obtained by SCA, HHO, AOA, SOA, STOA, AO, and ChOA algorithms. Each algorithm would also be run for 20 times and Wilcoxon Rank Sum test would also be calculated.

### 5.1 Gear design problem

The gear design problem is a well-known engineering design problem [69]. The problem is that the cost of the gear ratio is minimized by optimizing the number of teeth of the gear. This question is unconstrained.

Consider:

$$\vec{x}=[x_{1}\;x_{2}\;x_{3}\;x_{4}]$$

Objective:

$$f(\vec{x})={\left(\frac{1}{6.913}-\frac{x_{2}x_{3}}{x_{1}x_{4}}\right)}^{2}$$

Variable ranges:

$$12{\leq x}_{1},x_{2},x_{3},x_{4}\leq 60$$

The experimental results as shown in Table 15, show that the CSCAHHO algorithm can obtain the best value in the design of gear. By observing the value of P, it can be seen that the results obtained by the CSCAHHO algorithm are significantly different from other algorithms.

**Table 15:** Algorithm comparison results of gear design problem.

Algorithm	x_1_	x_2_	x_3_	x_4_	Best	P-value
**CSCAHHO**	**49**	**19**	**16**	**43**	**2.7009e-12**	**NaN**
SCA	60	20	26	60	2.7265e-08	1.2290e-05
HHO	43	19	16	49	2.7009e-12	0.0075
AOA	54	12	37	57	8.8876e-10	0.0013
SOA	43	19	16	49	2.7009e-12	0.0230
STOA	53	13	30	51	2.3078e-11	0.0498
AO	53	15	26	51	2.3078e-11	0.0018
ChOA	47	12	26	46	9.9216e-10	0.0480

### 5.2 Welded beam design problem

The purpose of the welded beam design problem is to reduce the manufacturing cost of the design, and its essence is to minimize the cost problem. The purpose of the welded beam design problem is to reduce the manufacturing cost of the design, and its essence is to minimize the cost problem. This question involves four variables: weld thickness (h), the length (l), height (t), and weld thickness (h) of the bar. This question contains 7 constraints.

Consider:

$$\vec{x}=[x_{1}\;x_{2}\;x_{3}\;x_{4}]=[h\;l\;t\;b]$$

Objective:

$$f(\vec{x})=1.10471x_{2}x_{1}^{2}+0.04811x_{3}x_{4}(14.0+x_{2})$$

Subject to:

$$g_{1}(\vec{x})=\tau (\vec{x})-\tau_{max}\leq 0$$

$$g_{2}(\vec{x})=\sigma (\vec{x})-\sigma_{max}\leq 0$$

$$g_{3}(\vec{x})=\delta (\vec{x})-\delta_{max}\leq 0$$

$$g_{4}(\vec{x})=x_{1}-x_{4}\leq 0$$

$$g_{5}(\vec{x})=P-P_{C}(\vec{x})\leq 0$$

$$g_{6}(\vec{x})=0.125-x_{1}\leq 0$$

$$g_{7}(\vec{x})=1.10471x_{1}^{2}+0.04811x_{3}x_{4}(14.0+x_{2})-5.0\leq 0$$

Variable ranges:

$$0.1\leq x_{1},x_{4}\leq 2$$

$$0.1\leq x_{2},x_{3}\leq 10$$

where:

$$\tau (\vec{x})=\sqrt{{\left(\tau^{\prime }\right)}^{2}+2\tau^{\prime }\tau^{\prime \prime }\frac{x_{2}}{2R}+{\left(\tau^{\prime \prime }\right)}^{2}}$$

$$\tau^{\prime }=\frac{P}{\sqrt{2}x_{1}x_{2}},\tau^{\prime \prime }=\frac{MR}{J},M=P(L+\frac{x_{2}}{2})$$

The experimental results as shown in Table 16, show that the CSCAHHO algorithm can obtain the best results, and there is a difference between the results of the comparison algorithm.

**Table 16:** Algorithm comparison results of welded beam design problem.

Algorithm	X_1_	X_2_	X_3_	X_4_	Best	P-value
**CSCAHHO**	**0.18477**	**3.6225**	**9.1981**	**0.20851**	**1.7627**	**NaN**
SCA	0.2657	4.8499	6.6577	0.51786	3.5049	1.0335e-04
HHO	0.15006	4.6094	9.0707	0.20659	1.7924	1.8901e-04
AOA	0.168106	4.24431	10	0.228865	2.1413	6.8061e-04
SOA	0.154029	4.1843	10	0.225867	2.0857	0.0111
STOA	0.175084	4.29697	10	0.209822	1.9925	0.0206
AO	0.19997	3.3786	9.6892	0.20272	1.7915	0.005371093750000
ChOA	0.204639	3.03208	10	0.206578	1.833	4.4934e-04

### 5.3 Compression spring design problem

The problem of compression spring design is a well-known problem in mechanical engineering. The purpose of this problem is to minimize the weight of the tension/compression spring. This question contains three variables: wire diameter (d), average coil diameter (D), and effective number of coils (N). There are also 4 constraints.

Consider:

$$\vec{x}=[x_{1}\;x_{2}\;x_{3}]=[d\;D\;N]$$

Objective:

$$f(\vec{x})=(x_{3}+2)x_{2}x_{1}^{2}$$

Subject to:

$$g_{1}(\vec{x})=1-\frac{x_{2}^{3}x_{3}}{71785x_{1}^{4}}\leq 0$$

$$g_{2}(\vec{x})=\frac{4x_{2}^{2}-x_{1}x_{2}}{12566(x_{2}x_{1}^{3}-x_{1}^{4})}+\frac{1}{5108x_{1}^{2}}-1\leq 0$$

$$g_{3}(\vec{x})=1-\frac{140.45x_{1}}{x_{2}^{2}x_{3}}\leq 0$$

$$g_{4}(\vec{x})=\frac{x_{1}+x_{2}}{1.5}-1\leq 0$$

Variable ranges:

$$0.05{\leq x}_{1}\leq 2.00$$

$$0.25{\leq x}_{2}\leq 1.30$$

$$2.00{\leq x}_{3}\leq 15.00$$

The experimental results are shown in Table 17. The CSCAHHO algorithm obtained the best value. Compared with the comparison algorithm, the results obtained by CSCAHHO are significantly different.

**Table 17:** Algorithm comparison results of compression spring design problem.

Algorithm	X_1_	X_2_	X_3_	Best	P-value
**CSCAHHO**	**0.0505056**	**0.32893**	**13.242**	**0.012789**	**NaN**
SCA	0.06229	0.51935	9.1949	0.022559	8.8575e-05
HHO	0.05522	0.44784	7.4308	0.012881	0.0333
AOA	0.05	0.310404	15	0.013193	0.0228
SOA	0.058344	0.53848	5.3525	0.013477	1.8901e-04
STOA	0.05	0.05	14.176	0.012802	8.8575e-05
AO	0.05	0.315738	15	0.013419	4.4934e-04
ChOA	0.05	0.317185	14.1908	0.012839	0.0051

## 6 Discussions and conclusions

In this paper, a chaotic hybridization algorithm of Sine Cosine algorithm (SCA) and Harris Hawk optimization algorithm (HHO) are proposed. This algorithm extracts the exploration capabilities of the improved SCA algorithm and the exploitation capabilities of the HHO algorithm, and then these two capabilities are mixed. The improved SCA algorithm adds flight operators and tends to the global optimum, which improves the global search capability of the SCA algorithm. Inspired by the phase transition in the HHO algorithm, its control parameter E is introduced. In order to increase the randomness in the control parameter, a chaotic map is introduced. In order to evaluate the performance of the CSCAHHO algorithm accurately, 27 standard functions and CEC2014 benchmark functions and three engineering design questions were tested. The experimental results are compared with other meta-heuristic algorithms, which prove the CSCAHHO algorithm has better global exploration capabilities, faster convergence speed, and higher convergence accuracy.

Although the proposed CSCAHHO algorithm is a hybridization, however, the detailed simulation experiments carried out in this paper verified its better performance. We can see those multiple ways for individuals to update their equations, or chaotic improvements, even the energy parameter balancing the exploration and exploitation procedure during iterations, play an important role in improving the capability of the existed algorithms. Hybridization algorithms would be more efficient in optimization. How to made an easy hybridization of the existed algorithms with fast convergence, low residual errors, stability and steadiness, might be a promising work in the future.
